# Model-based assessment of replicability for genome-wide association meta-analysis

**DOI:** 10.1038/s41467-021-21226-z

**Published:** 2021-03-30

**Authors:** Daniel McGuire, Yu Jiang, Mengzhen Liu, J. Dylan Weissenkampen, Scott Eckert, Lina Yang, Fang Chen, GWAS and Sequencing Consortium of Alcohol and Nicotine Use (GSCAN), Mengzhen Liu, Yu Jiang, Robbee Wedow, Yue Li, David M. Brazel, Fang Chen, Gargi Datta, Jose Davila-Velderrain, Daniel McGuire, Chao Tian, Xiaowei Zhan, H. éléne Choquet, Anna R. Docherty, Jessica D. Faul, Johanna R. Foerster, Lars G. Fritsche, Maiken Elvestad Gabrielsen, Scott D. Gordon, Jeffrey Haessler, Jouke-Jan Hottenga, Hongyan Huang, Seon-Kyeong Jang, Philip R. Jansen, Yueh Ling, Reedik Ma ¨gi, Nana Matoba, George McMahon, Antonella Mulas, Valeria Orru, Teemu Palviainen, Anita Pandit, Gunnar W. Reginsson, Anne Heidi Skogholt, Jennifer A. Smith, Amy E. Taylor, Constance Turman, Gonneke Willemsen, Hannah Young, Kendra A. Young, Gregory J. M. Zajac, Wei Zhao, Wei Zhou, Gyda Bjornsdottir, Jason D. Boardman, Michael Boehnke, Dorret I. Boomsma, Chu Chen, Francesco Cucca, Gareth E. Davies, Charles B. Eaton, Marissa A. Ehringer, To ~nu Esko, Edoardo Fiorillo, Nathan A. Gillespie, Daniel F. Gudbjartsson, Toomas Haller, Kathleen Mullan Harris, Andrew C. Heath, John K. Hewitt, Ian B. Hickie, John E. Hokanson, Christian J. Hopfer, David J. Hunter, William G. Iacono, Eric O. Johnson, Yoichiro Kamatani, Sharon L. R. Kardia, Matthew C. Keller, Manolis Kellis, Charles Kooperberg, Peter Kraft, Kenneth S. Krauter, Markku Laakso, Penelope A. Lind, Anu Loukola, Sharon M. Lutz, Pamela A. F. Madden, Nicholas G. Martin, Matt McGue, Matthew B. McQueen, Sarah E. Medland, Andres Metspalu, Karen L. Mohlke, Jonas B. Nielsen, Yukinori Okada, Ulrike Peters, Tinca J. C. Polderman, Danielle Posthuma, Alexander P. Reiner, John P. Rice, Eric Rimm, Richard J. Rose, Valgerdur Runarsdottir, Michael C. Stallings, Alena Stanˇca ´kova, Hreinn Stefansson, Khanh K. Thai, Hilary A. Tindle, Thorarinn Tyrfingsson, Tamara L. Wall, David R. Weir, Constance Weisner, John B. Whitfield, Bendik Slagsvold Winsvold, Jie Yin, Luisa Zuccolo, Laura J. Bierut, Kristian Hveem, James J. Lee, Marcus R. Munafo, Nancy L. Saccone, Cristen J. Willer, Marilyn C. Cornelis, Sean P. David, David Hinds, Eric Jorgenson, Jaakko Kaprio, Jerry A. Stitzel, Kari Stefansson, Thorgeir E. Thorgeirsson, Goncalo Abecasis, Dajiang J. Liu, Scott Vrieze, Arthur Berg, Scott Vrieze, Bibo Jiang, Qunhua Li, Dajiang J. Liu

**Affiliations:** 1 Department of Public Health Sciences, Penn State College of Medicine, Hershey, PA USA; 2 Department of Psychology, University of Minnesota, Minneapolis, MN USA; 3 Department of Statistics, Penn State University, University Park, PA USA; 4 Department of Psychology, University of Minnesota Twin Cities, Minneapolis, MN USA; 5 Department of Public Health Sciences, College of Medicine, Pennsylvania State University, Hershey, PA USA; 6 Institute of Personalized Medicine, College of Medicine, Pennsylvania State University, Hershey, PA USA; 7 Institute for Behavioral Genetics, University of Colorado Boulder, Boulder, CO USA; 8 Department of Sociology, University of Colorado Boulder, Boulder, CO USA; 9 Institute of Behavioral Science, University of Colorado Boulder, Boulder, CO USA; 10 Computer Science and Artificial Intelligence Lab, Massachusetts Institute of Technology, Cambridge, MA USA; 11 The Broad Institute of MIT and Harvard, Cambridge, MA USA; 12 Department of Molecular, Cellular, and Developmental Biology, University of Colorado Boulder, Boulder, CO USA; 13 Interdisciplinary Quantitative Biology Graduate Group, University of Colorado Boulder, Boulder, CO USA; 14 23andMe, Inc., Mountain View, CA USA; 15 Quantitative Biomedical Research Center, Department of Clinical Sciences, University of Texas Southwestern Medical Center, Dallas, TX USA; 16 Center for the Genetics of Host Defense, Department of Clinical Sciences, University of Texas Southwestern Medical Center, Dallas, TX USA; 17 Division of Research, Kaiser Permanente Northern California, Oakland, CA USA; 18 Department of Psychiatry, Virginia Institute for Psychiatric & Behavioral Genetics, Virginia Commonwealth University, Richmond, VA USA; 19 Department of Psychiatry and Human Genetics, University of Utah, Salt Lake City, UT USA; 20 Survey Research Center, Institute for Social Research, University of Michigan, Ann Arbor, MI USA; 21 Department of Biostatistics, Center for Statistical Genetics, University of Michigan, Ann Arbor, MI USA; 22 K.G. Jebsen Center for Genetic Epidemiology, Department of Public Health and Nursing, Norwegian University of Science and Technology, Trondheim, Norway; 23 Genetic Epidemiology, QIMR Berghofer Medical Research Institute, Brisbane, QLD Australia; 24 Division of Public Health Sciences, Fred Hutchinson Cancer Research Center, Seattle, WA USA; 25 Department of Biology Psychology, Vrije Universiteit Amsterdam, Amsterdam, The Netherlands; 26 Program in Genetic Epidemiology and Statistical Genetics, Harvard T.H. Chan School of Public Health, Boston, MA USA; 27 Department of Epidemiology, Harvard T.H. Chan School of Public Health, Boston, MA USA; 28 Department of Complex Trait Genetics, Center for Neurogenomics and Cognitive Research, Vrije Universiteit Amsterdam, Amsterdam, The Netherlands; 29 Department of Child and Adolescent Psychiatry, Erasmus MC Rotterdam, Rotterdam, The Netherlands; 30 Estonian Genome Center, University of Tartu, Tartu, Estonia; 31 Laboratory for Statistical Analysis, RIKEN Center for Integrative Medical Sciences, Yokohama City, Kanagawa Japan; 32 Department of Population Health Science, Bristol Medical School, Oakfield Grove, Bristol, UK; 33 Consiglio Nazionale delle Ricerche, Istituto di Ricerca Genetica e Biomedica, Monserrato, Italy; 34 Institute for Molecular Medicine Finland (FIMM), University of Helsinki, Helsinki, Finland; 35 deCODE Genetics/AMGEN, Inc., Reykjavik, Iceland; 36 Department of Epidemiology, University of Michigan, Ann Arbor, MI USA; 37 Department of Epidemiology, University of Colorado Anschutz Medical Campus, Aurora, CO USA; 38 Department of Computational Medicine and Bioinformatics, University of Michigan, Ann Arbor, MI USA; 39 Avera Institute for Human Genetics, Sioux Falls, SD USA; 40 Department of Family Medicine & Community Health, Alpert Medical School, Brown University, Providence, RI USA; 41 Department of Integrative Physiology, University of Colorado Boulder, Boulder, CO USA; 42 School of Engineering and Natural Sciences, University of Iceland, Reykjavik, Iceland; 43 Department of Sociology, University of North Carolina at Chapel Hill, Chapel Hill, NC USA; 44 Carolina Population Center, University of North Carolina at Chapel Hill, Chapel Hill, NC USA; 45 Department of Psychiatry, Washington University in St. Louis, St. Louis, MO USA; 46 Department of Psychology and Neuroscience, University of Colorado Boulder, Boulder, CO USA; 47 Brain and Mind Centre, University of Sydney, Sydney, New South Wales Australia; 48 Department of Psychiatry, University of Colorado Anschutz Medical Campus, Aurora, CO USA; 49 Nuffield Department of Population Health, University of Oxford, Oxford, UK; 50 Fellows Program, RTI International, Research Triangle Park, NC USA; 51 Department of Biostatistics, Harvard T.H. Chan School of Public Health, Boston, MA USA; 52 Department of Internal Medicine, Institute of Clinical Medicine, University of Eastern Finland, Kuopio, Finland; 53 Department of Medicine, Kuopio University Hospital, Kuopio, Finland; 54 Psychiatric Genetics, QIMR Berghofer Medical Research Institute, Brisbane, QLD Australia; 55 Department of Biostatistics and Bioinformatics, University of Colorado Anschutz Medical Campus, Aurora, CO USA; 56 Department of Genetics, University of North Carolina at Chapel Hill, Chapel Hill, NC USA; 57 Department of Internal Medicine, Division of Cardiovascular Medicine, University of Michigan, Ann Arbor, MI USA; 58 Department of Statistical Genetics, Osaka University Graduate School of Medicine, Suita, Osaka, Japan; 59 Department of Epidemiology, University of Washington, Seattle, WA USA; 60 Department of Clinical Genetics, VU Medical Centre Amsterdam, Amsterdam, The Netherlands; 61 Department of Psychiatry, Washington University School of Medicine, St. Louis, MO USA; 62 Department of Nutrition, Harvard T.H. Chan School of Public Health, Boston, MA USA; 63 Department of Psychological and Brain Sciences, Indiana University, Bloomington, IN USA; 64 SAA - National Center of Addiction Medicine, Vogur Hospital, Reykjavik, Iceland; 65 Department of Medicine, Vanderbilt University, Nashville, TN USA; 66 Department of Psychiatry, University of California San Diego, San Diego, CA USA; 67 FORMI and Department of Neurology, Oslo University Hospital, Oslo, Norway; 68 MRC Integrative Epidemiology Unit, University of Bristol, Oakfield Grove, Bristol, UK; 69 HUNT Research Centre, Department of Public Health and Nursing, Norwegian University of Science and Technology, Levanger, Norway; 70 Department of Medicine, Levanger Hospital, Nord-Trøndelag Hospital Trust, Levanger, Norway; 71 UK Centre for Tobacco and Alcohol Studies, School of Psychological Science, University of Bristol, Bristol, UK; 72 Department of Genetics, Washington University School of Medicine, St. Louis, MO USA; 73 Department of Human Genetics, University of Michigan, Ann Arbor, MI USA; 74 Northwestern University Feinberg School of Medicine, Department of Preventative Medicine, Chicago, IL USA; 75 Department of Medicine, Stanford University School of Medicine, Stanford, CA USA; 76 Department of Public Health, University of Helsinki, Helsinki, Finland; 77 Faculty of Medicine, University of Iceland, Reykjavik, Iceland

**Keywords:** Software, Genome-wide association studies, Genomics, Statistics

## Abstract

Genome-wide association meta-analysis (GWAMA) is an effective approach to enlarge sample sizes and empower the discovery of novel associations between genotype and phenotype. Independent replication has been used as a gold-standard for validating genetic associations. However, as current GWAMA often seeks to aggregate all available datasets, it becomes impossible to find a large enough independent dataset to replicate new discoveries. Here we introduce a method, MAMBA (Meta-Analysis Model-based Assessment of replicability), for assessing the “posterior-probability-of-replicability” for identified associations by leveraging the strength and consistency of association signals between contributing studies. We demonstrate using simulations that MAMBA is more powerful and robust than existing methods, and produces more accurate genetic effects estimates. We apply MAMBA to a large-scale meta-analysis of addiction phenotypes with 1.2 million individuals. In addition to accurately identifying replicable common variant associations, MAMBA also pinpoints novel replicable rare variant associations from imputation-based GWAMA and hence greatly expands the set of analyzable variants.

## Abstract (web summary)

In genome-wide association meta-analysis, it is often difficult to find an independent dataset of sufficient size to replicate associations. Here, the authors have developed MAMBA to calculate the probability of replicability based on consistency between datasets within the meta-analysis.

## Introduction

Genome wide association meta-analysis (GWAMA) is an effective approach to enlarge sample size and empower the discovery of genetic variants associated with complex traits. In the past decade, GWAMA identified numerous genetic variants that are associated with various complex traits, including cardiovascular diseases^1,2^, diabetes^3^, and cancer^4,5^. These associated variants helped narrow down the list of potential causal genes, and provided numerous targets for biological follow-up and drug development^6–8^. For the years to come, it will be a central focus of disease biology to understand the functional and clinical consequences of GWAS loci.

A critical step preceding any functional follow-up is to confirm the validity of the identified association signals. Ascertainment bias, phenotyping or genotyping error, population structure, or cryptic relatedness can all cause false positive discoveries and mislead downstream functional studies that are costly to perform. To minimize false positive findings, replication is often conducted using an independent dataset. If the identified association remains significant, the signal is considered as replicated and likely valid. While replication is the gold standard for validating GWAS discovery, there is always a tension between the motivation of designating a suitably sized replication dataset, and aggregating all available cohorts in a discovery sample to maximize the power of genetic discovery. Just as in discovery samples, replication studies can also have type I or type II errors, so it is important that replication studies should be of sufficient sample size to convincingly distinguish the non-zero effect from the null effect^9^. As GWAS discovery sample sizes increase, newly identified loci tend to have smaller effect sizes, or come from variants with rare minor allele frequency^10^, which makes finding a sufficiently powered replication dataset increasingly challenging. Moreover, after replication, studies often seek to jointly analyze the discovery and replication datasets to discover additional loci, which will be left unreplicated. As such, there is a compelling need to develop a principled statistical model-based approach to assess the replicability of genetic association studies when a suitable replication dataset is unavailable.

Classical approaches for meta-analysis, such as fixed effects^11^, random effect meta-analysis^12^, or their adaptations in GWAS^13,14^, do not specifically address the replicability problem. These methods may produce spurious meta-analysis results when some participating studies contain false positive signals. In practice, some ad hoc procedures may be applied to examine the validity of the results^15^, e.g. if the association signal is supported by a certain number of participating studies or if the heterogeneities of the genetic effect between genetically similar populations are small^16^, which can be hard to reproduce and generalize. Also, in order to protect against spurious associations, some overly conservative criteria may be applied in the quality control, e.g. studies may attempt to remove all low-frequency variants from imputation-based GWAS^17^, even though many of the imputed low-frequency variants may still be informative and causative. Some principled methods exist for assessing the replicability for biological experiments, including *repfdr* and *SCREEN* which were developed specifically for GWAMA^18–20^. These existing methods seek to leverage the strength and consistency of the signals between biological replicates to distinguish replicable and non-replicable signals. Yet there are several limitations to these approaches when applied to GWAMA. For one, they only rely on the statistical significance of the association but do not consider the estimated effect sizes, or the potential sample size differences between participating studies. Large datasets produce more significant p-values compared to smaller studies when the estimated association effect size (either genuine or spurious) is the same, so the significance of association in each cohort is not a reliable measure for replicability. Also, some of these methods (e.g. *repfdr*^18^) were developed for a few biological replicates and cannot scale well with meta-analyses with many participating studies.

We address the limitations of existing methods by developing a principled approach MAMBA (Meta-Analysis Model-based Assessment of replicability) to assess the replicability of GWAMA association signals. Our approach models the genetic effects as a mixture of SNPs with real non-zero effects, normally-behaved null SNPs, and SNPs that have null effects but appear as spurious association signals in some participating studies due to artifacts in the data. MAMBA performs meta-analysis for genome-wide SNPs and calculates a posterior probability of replicability (PPR) that a given SNP has a non-zero replicable effect. Similar to other methods for assessing replicability, our method exploits cohort-level summary association statistics from multiple studies in GWAMA. It assigns a higher PPR to an association signal, if the SNP is significantly associated with the phenotype and its estimated effect sizes are consistent across multiple studies. Compared to other meta-analysis methods, MAMBA is much more robust to outlier studies. In the special case that fixed effects assumptions hold, and no heterogeneity or outliers are present, MAMBA is similar to a standard inverse-variance weighted meta-analysis (except that MAMBA imposes a prior on the distribution of effect sizes across SNPs), resulting in virtually no loss of power compared to the widely used fixed effect meta-analysis. We conduct extensive simulations to evaluate the performance of our approach in assessing the replicability of association signals in meta-analysis across a wide range of scenarios. We show that MAMBA can powerfully identify replicable association signals. It also improves the genetic effect estimates by borrowing information across genome-wide SNPs and applying shrinkage. We further demonstrate the value of the method by applying it to a GWAMA of several smoking and drinking addiction phenotypes from the GWAS and Sequencing Consortium of Alcohol and Nicotine use (GSCAN), where summary statistics are aggregated from 35 individual study cohorts of European ancestry, and up to 1.2 million research participants^17^. In the published meta-analysis^17^, a stringent quality control was conducted and only variants with MAF > 0.1% were analyzed to ensure the quality of the results, yet it potentially left out well-imputed rare frequency variants with MAF < 0.1%. In this study, we reanalyze the common variants (with MAF > 1%) and low-frequency variants (0.1%<MAF < 1%) analyzed in the original study, as well as the rare imputed variants (with MAF < 0.1%) using MAMBA. Among the 556 published common and low-frequency variant signals, we identify only one with low PPR (<10%), while 529 have PPR greater than 99%. In our extended analysis of ~4300 rare imputed variants, we identify 2,807 variants with PPR greater than 99% with many being coding variants. These identified rare variant association signals pinpoint potential new loci with pleiotropic effects on lipids metabolisms, immunity, and substance use. MAMBA hence further expands the utility of imputation-based genetic studies to robustly study rare variants.

In this work, summarily, we propose methods for assessing replicability from GWAMA, reanalyze an ultra-large-scale GWAMA of tobacco and alcohol use phenotype, and identify a number of interesting rare variant associations. The proposed methods and software will benefit future large-scale genetic studies using biobanks.

## Results

### A motivating example

**Fig. 1:**
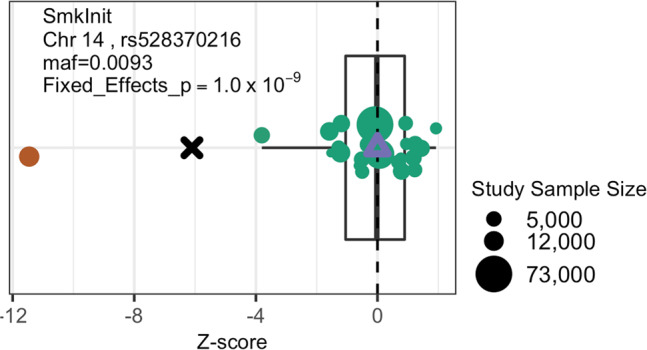
Cohort level Z-scores for a genome-wide significant SNP that was identified as non-replicable for a Smoking Initiation (SmkInit) phenotype from GSCAN Consortium. Cross-mark indicates the meta-analysis z-score from a two-sided hypothesis test unadjusted for multiple comparisons. The size of the dot is proportional to the sample size of the cohort. Identified “outlier” summary statistic is marked as orange, which has a disproportionally large Z-score and drives the meta-analysis association results. The purple triangle indicates the MAMBA estimated Z-score, the posterior probability of replicability is 9 × 10^−22^, very close to 0. Source data are provided as a Source Data file.

The MAMBA model was motivated by the observed patterns of outliers from multiple large-scale GWAMA on lipids levels and smoking drinking traits. As a motivating example, we plotted the contributed summary association statistics (i.e. the Z-score statistic) from each participating study (Fig. 1) for a SNP for a Smoking Initiation (SmkInit) phenotype in GSCAN. Under the assumption that the genetic effects are similar in different studies, the magnitude of the Z-score statistic should be approximately proportional to the square root of the sample size. However, as shown in Fig. 1, there is an outlier study that contributes a disproportionally large Z-score, which leads to a significant fixed effect meta-analysis*p*-value (*p* = 1 × 10^−9^). Just as in this example, an outlier from a contributing study may easily dominate the result in a fixed effect meta-analysis, even if a majority of the test statistics follow the null distribution. This insight motivated us to model the effect size estimates from participating studies as a mixture of outliers with inflated variance and normal well-behaved estimates.

### Methods overview

MAMBA is a two-level mixture model that takes the genetic effect ***b***_*j*_ and its standard deviation ***s***_*j*_ from participating studies as input for a particular SNP ***j***, i.e. ${\boldsymbol{b}}_{\boldsymbol{j}} = ( {b_{j1}, \ldots ,b_{jK}} )$ and ${\boldsymbol{s}}_{\boldsymbol{j}} = ( {s_{j1}, \ldots ,s_{jK}})$. To mathematically describe this model, we define indicator variable *R*_*j*_ ~ Bernoulli (*π*), with *R*_*j*_ = 1 if the SNP has a real non-zero effect. When the SNP has null effect (i.e. *R*_*j*_ = 0), we further define an indicator variable *O*_*jk*_ ~ Bernoulli (*λ*) indicating whether the SNP is a spurious association with inflated variation (i.e. *O*_*jk*_ = 1) in study *k*, or it is a well-behaved null SNP (i.e. *O*_*jk*_ = 0). Conditional on the indicators, the distribution of the genetic effect *b*_*jk*_ satisfies

1 $$\begin{array}{l}p(b_{jk}|\mu _j,R_j = 1)\sim N\big( {\mu _j,s_{jk}^2} \big)\;{\mathrm{with}}\; p\big( {\mu _j|R_j = 1} \big)\sim N\left( {0,\tau ^2} \right)\\ p\big( {b_{jk}|\mu _j = 0,R_j = 0,O_{jk} = 0} \big)\sim N\big( {0,s_{jk}^2} \big)\\ p(b_{jk}|\mu _j = 0,R_j = 0,O_{jk} = 1)\sim N\big( {0,\alpha s_{jk}^2} \big)\end{array}$$

Here *a* is an inflation factor which captures the extent of inflation in the observed effect sizes for outlier summary statistics (i.e. *R*_*j*_ = 0, *O*_*jk*_ = 1). As a special case, when no outliers exist, conditional on the mean value parameter *μ*_*j*_, the MAMBA model reduces to that of a fixed effect meta-analysis^21^, i.e. $p(b_{jk}|\mu _j)\sim N( {\mu _j,s_{jk}^2} )$ for all SNPs. As the goal of the model is to identify replicable associations, we do not allow for outliers or model variance inflation when a SNP has real non-zero genetic effect (i.e. the case *R*_*j*_ =1, *O*_*jk*_ = 1 is not considered in our model). To assess the replicability of GWAS loci, we choose the sentinel variant from each locus as input, which is pruned based upon linkage disequilibrium. We assume that the SNPs used in the model are independent, so the likelihood for all SNPs becomes the product of the likelihood of individual SNPs. When it is of interest to estimate the genetic effect sizes of all variants in a locus (and not merely the sentinel variants), we found that fitting the same model using correlated SNPs led to similar improvements as MAMBA in estimating genetic effect size. In this case, the model can be considered as a composite likelihood (MAMBA-est), which takes all SNPs in the identified loci as input. This allows for genome-wide estimates of genetic effect size, which can be used for many downstream analyses. If the primary goal of the analysis is assessing replicability, MAMBA is preferred to MAMBA-est due to its computational convenience. A more detailed comparison of MAMBA and MAMBA-est can be found in “Results”.

Using an expectation-maximization algorithm, we estimate the hyperparameters from the data, and calculate the PPR. MAMBA (and MAMBA-est) give improved estimates of the genetic effect by modeling the joint distribution of the effect sizes across different genetic variants. To facilitate the comparison with frequentist methods, we further developed a parametric bootstrap method to calculate p-values testing *H*_0_:*μ*_*j*_ = 0 for each SNP. More model details can be found in the “Online Methods”.

### Simulation studies

We conducted extensive simulations to compare the performance of MAMBA with existing meta-analysis and replicability analysis methods. We assessed the models in terms of type I error control, power, and estimation of the underlying effect size. The models considered for comparison include

1. fixed effects inverse variance weighted meta-analysis (FE);
2. random effects DerSimonian-Laird model (RE)^12^;
3. Han and Eskin’s random effects model (RE2) that assumes no heterogeneity across studies under the null hypothesis^13^;
4. Binary effects model (BE)^14^ that assumes for each SNP, a portion of the studies in the meta-analysis have null effects while the rest of the studies have fixed effects;
5. SCREEN method for replicability analysis^19^, a method which calculates the posterior probability that a SNP has non-zero effect in at least a given number of studies.

As each method makes different assumptions regarding the distribution of the estimated effect sizes across studies, we considered 5 different data generation processes (DGP) to facilitate a comprehensive and fair comparison between different methods. Under each DGP, we simulated 60 million independent SNPs, and randomly picked 1% of SNPs to have true non-zero effect, which are normally distributed with mean zero and variance *τ*^2^. The effect size estimate variances $s_{jk}^2$ were generated in our simulation by sampling with replacement from the variance of the observed genetic effects calculated from existing GSCAN study summary statistics. The effect size estimates were then simulated based upon the estimated true effect sizes and standard errors sampled from the GSCAN studies, following the assumptions of each DGP.

For the MAMBA DGP, we varied the severity of outlier test statistics, while for RE and RE2 DGP we varied the amount of effect heterogeneity across cohorts. For FE DGP we considered different magnitudes of fixed effects sizes. For BE DGP we randomly selected a fraction of the studies where the genetic effect of causal variants is non-zero. A complete and detailed breakdown of simulation scenarios considered can be found in the Supplementary Note.

#### Simulation evaluation of type I error

We evaluated empirical type-I error rates at α = 1 × 10^−6^, 1 × 10^−5^, 1 × 10^−3^, and 0.05 for each method under different DGPs (Supplementary Data 1). The type I error was evaluated using 60 million simulated genetic variants for each DGP.

First, we found that under the fixed effects assumption (DGP=FE), all methods have controlled type I error for different significance thresholds, except for the RE method which tends to be conservative. When outliers are present in the dataset (DGP=MAMBA), all models except for MAMBA have inflated type-I error. The inflation of the type-I error rate becomes increasingly severe as the significance level becomes more stringent. For example, at α = 1 × 10^−3^, type I error for the FE method is 5 times inflated relative to the significance threshold, and BE and RE2 methods are both >10 times inflated. At a more stringent threshold of α = 1 × 10^−6^, the type I error for the FE method is >400 times the significance threshold, whereas BE and RE2 both have type-I error rates of more than 4000 times the significance threshold.

Interestingly, the RE method does not have well-controlled type-I error even when the data are generated under a RE DGP. This is in fact consistent with previous investigations^22–24^. The type I error inflation is due to the challenge of accurately estimating the heterogeneity in a set of meta-analysis studies. On the other hand, the MAMBA model produces better-calibrated p-values compared to the RE model even when the data is generated according to a RE model. For example, at α = 1 × 10^−6^, the RE method type-I error rate is 9.7 × 10^−6^, close to 10 times the significance threshold, while the type I errors for FE, RE2, and BE methods are all greater than 20 times the nominal threshold. The SCREEN model was not considered here, as it does not calculate meta-analysis*p*-values.

#### Simulation comparison of power

**Fig. 2:**
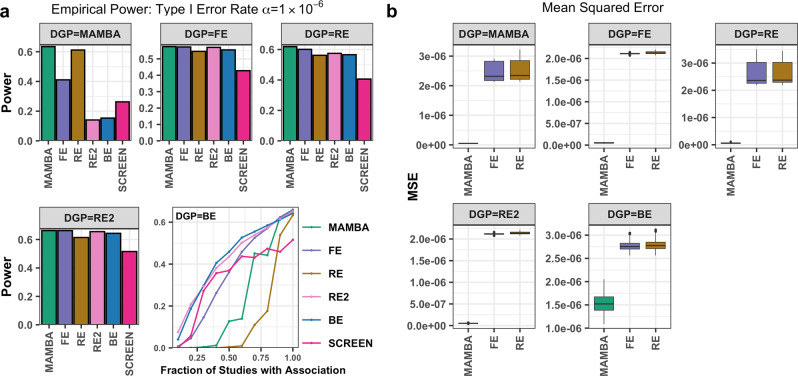
Comparison of power and the mean square error for different methods in simulation studies. **a** The power was evaluated using 1 million replications at the significance threshold of α = 1 × 10^−6^. **b** The mean-squared error for the genetic effect estimates were evaluated for MAMBA, fixed effect (FE) and random effect meta-analysis (RE). The RE2, BE, and SCREEN methods do not produce genetic effect estimates and thus were not considered. Scenarios under different data generation process (DGP) were considered. Source data are provided as a Source Data file.

We next compared each method in terms of power under different DGPs (Fig. 2a). As some methods have inflated type-I error rates, we recalibrated the significance threshold for each method so that all methods have an empirical type-I error rate α = 1 × 10^−6^. The power comparison was based upon the recalibrated threshold (Supplementary Data 2). We first note that when standard fixed-effects assumptions hold (DGP=FE), power for the MAMBA model is nearly equal to that for fixed-effects meta-analysis, and larger than any alternative methods. When the data are generated with outliers or heterogeneity (DGP=MAMBA or RE DGP), the power of the MAMBA model is also greater than that of any other method. Under an RE2 DGP, where heterogeneity exists only under the alternative hypothesis, MAMBA and FE have nearly identical power, and both are slightly more powerful than the RE2 method. This comparison is in fact consistent with Han and Eskin’s finding^13^, and is reflective of the amount of between-study heterogeneity (0.05–0.3) we used in the simulation studies. In general, one would expect some advantages for the RE2 method over alternatives in cases of more extreme effect heterogeneity. Finally, while the BE model has superior power when less than 90% of the studies are associated with the phenotype, the MAMBA and FE models are the most powerful methods when the genetic variant is associated with the phenotype in 90% or more of the studies. As the goal of the MAMBA model is to identify real and non-zero replicable associations where effects are present in all cohorts, the comparison result with BE is expected in cases where only a small proportion of studies are associated with the phenotype.

#### Improved accuracy for genetic effect estimates

In assessing the accuracy of effect size estimation, we observed that the MAMBA model exhibits lower mean-squared error (MSE) between the estimated and true effect sizes compared to FE or RE methods regardless of the DGP (Fig. 2b). This is likely because the MAMBA posterior estimator benefits from shrinkage achieved by jointly modeling all SNPs. Under the FE, RE, RE2, and MAMBA DGP, we noted that the MSEs of genetic effect estimates from FE and RE models are more than 40 times larger than that of the MAMBA model. The BE, RE2, and SCREEN models were not considered for comparison here as they do not directly estimate effect size.

#### Estimation of MAMBA Hyperparameters

A summary of hyperparameter estimates across all simulated DGP is shown in Supplementary Data 3. When the data are generated according to the MAMBA model, average estimates of MAMBA hyperparameters are close to the true values used in the simulation. Under a FE DGP, the inflation factor α converges to nearly 1, which indicates no inflation and is equivalent to the FE DGP assumption. We found that under RE or RE2 DGP with the *I*^2^ heterogeneity statistic between 5–30%, the fraction of estimated outlier studies was large (~0.6), but the estimate of variance inflation α was moderate (between 1 and 1.6). As indicated by well-controlled Type I error, MAMBA appears to be flexible enough to adequately model a RE DGP. Under all DGP, the estimated proportion and variance of replicable non-zero effect SNPs were well estimated by the MAMBA model. We also ran additional simulation scenarios, considering cases where the MAMBA inflation factor was large and the proportion of outliers was small (α = 100, *λ* = 0.001), and where the inflation factor is relatively modest (α = 1.1, *λ* = 0.025). These scenarios are reflective of the models estimated for GSCAN addiction phenotypes. We found that MAMBA hyperparameter estimates remained unbiased with well-controlled Type-I error rates, with power and MSE of effect sizes improved compared to alternative methods (Supplementary Data 4).

### Application to GSCAN meta-analysis of addiction phenotypes

We also used the GSCAN dataset to compare meta-analysis methods and their potential to assess replicability in GWAS. The GSCAN study consists of 35 contributing research studies and a combined sample size of up to 1.2 million participants^17^. In this study, a total of 406 novel loci were identified. Here, we consider analyzing Drinks per Week (DrnkWk), Smoking Initiation (SmkInit), Smoking Cessation (SmkCes), and Cigarettes Per Day (CigDay) phenotypes. Table 1 displays the sample sizes for each trait. More detailed information on the participating cohorts can be found in Supplementary Data 5–6. Minor allele frequencies from all variants in each GSCAN cohort were shared in meta-analysis, and the overall MAF was calculated across cohorts using the individual cohort MAFs. All GSCAN cohorts were of European ancestry. Participating studies in the meta-analysis were approved by their local Institutional Review Board.

**Table 1:** Sample size for discovery and replication cohorts for smoking and drinking phenotypes in GSCAN.

Phenotype	Discovery *N* (number of contributing studies)	Replication sample size (23andMe)
CigDay	263,954 (34)	73,380
DrnkWk	537,349 (33)	403,931
SmkCes	366,740 (36)	234,398
SmkInit	651,337 (35)	599,289

**Fig. 3:**
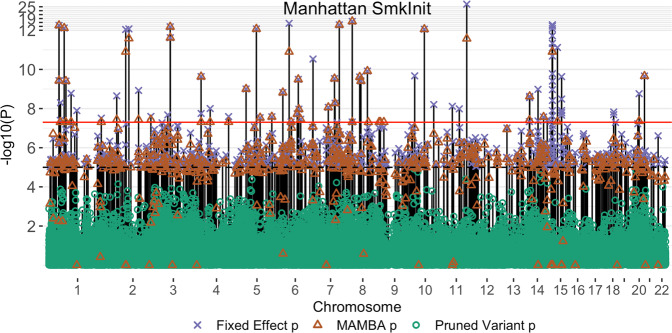
Layered Manhattan plot for smoking initiation (SmkInit) phenotype. Each vertical line represents a SNP analyzed by the MAMBA, where the line extends to a purple cross indicating the fixed-effects *p*-value. Orange triangles on the same line indicate the MAMBA *p*-values for the same SNP. Green points are the p-values for randomly pruned markers included in the MAMBA model to ensure that both non-replicable and replicable associations are incorporated. *P*-values for the MAMBA model were calculated through a bootstrap procedure. All *p*-values are two-sided and not adjusted for multiple comparisons. Source data are provided as a Source Data file.

To evaluate different methods, we treated the 23andMe dataset as the replication cohort as it is the largest contributing study. We performed discovery meta-analysis using the remaining cohorts. In this way, we ensure that both the discovery and replication cohorts have adequate sample sizes and power. For each phenotype, we first conducted a fixed effect inverse variance weighted meta-analysis combining the genetic effect estimates. We analyzed all SNPs which were imputed in at least four cohorts. Among variants with marginal *p*-values < 1 × 10^−5^, we applied clumping^25^ and retained the SNP with the most significant *p*-value in each locus, and removed any SNPs within 500kB that have an LD coefficient of > 0.1 with the sentinel variant^26^. These retained SNPs were combined with non-significant (i.e. *p*-value > 1 × 10^−5^) pruned variants with minor allele frequency (MAF) > 0.01 to fit the MAMBA model. The non-significant pruned variants are included in the dataset to ensure that the non-replicable mixture component of the MAMBA model is represented and can be accurately estimated. Their inclusion is for numerical considerations. We also applied MAMBA-est to all SNPs in identified loci. For both MAMBA and MAMBA-est, a separate model was estimated for each chromosome to allow the hyperparameters to vary across chromosomes. The average time for model convergence was less than 2 minutes for MAMBA models and less than 5 minutes for MAMBA-est (Supplementary Data 7). Estimated model parameters for all GSCAN models are shown in Supplementary Data 8–11. A layered Manhattan plot illustrating the results of the MAMBA method for SmkInit is displayed in (Fig. 3).

#### GSCAN Analysis Demonstrates that MAMBA is More Powerful and Robust Than Alternative Methods

We ranked the *p*-values in the discovery and replication cohort separately, with smaller *p*-values given lower numerical rank. In assessing whether a SNP has a replicable association, we expect that the p-values for replicable signals will be consistently highly ranked in both the discovery and replication cohorts, while spurious signals from the discovery cohort will likely become insignificant and low-ranked in the replication cohort. To compare different methods we used Kendall’s-tau^27^ to assess the concordance between *p*-values in discovery and replication phase.

**Fig. 4:**
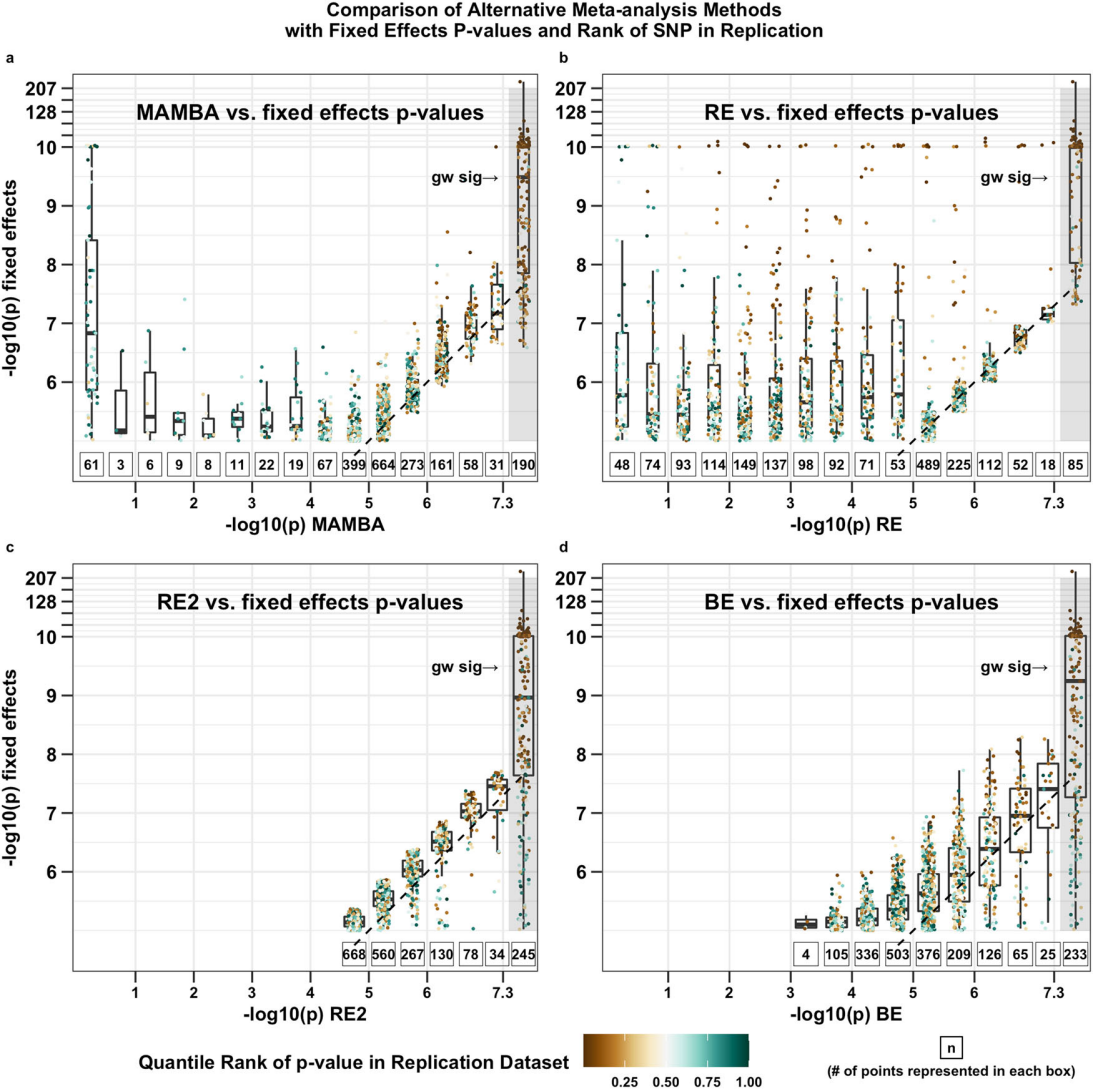
Comparison of the p-values from MAMBA, RE, RE2, and BE meta-analysis with that of a fixed effects meta-analysis combined across all GSCAN addiction phenotypes in the discovery cohorts. Each panel includes two-sided unadjusted p-values for 1,982 SNPs which were significant at *P* < 1 × 10^−5^ from a fixed effect meta-analysis and also tested in the replication dataset. We compare the p-values of fixed effect meta-analysis with those obtained from (**a**) MAMBA, (**b**) RE, (**c**) RE2, and (**d**) BE methods. The variants are colored by their p-values in the replication cohort, with brown dots indicating SNPs with the most significant replication p-values. The gray shaded region labeled ‘gw-sig’ indicates where the alternative method to fixed effects meta-analysis produces meta-analysis p-values < 5×10^−8^. The number of variants represented in each boxplot is denoted underneath the boxplot. Each boxplot denotes 25th percentile, median, and 75th percentiles with whiskers extending by 1.5 times the inter-quartile range below and above the 25th and 75th percentiles. For SNPs with more significant replication *p*-values, MAMBA produces similar results as fixed effect meta-analysis in the discovery cohorts. For SNPs with insignificant replication p-values, MAMBA produced much more conservative *p*-values in the discovery cohorts. Source data are provided as a Source Data file.

The p-values from both MAMBA and MAMBA-est had higher levels of concordance with the replication cohort p-value for every phenotype compared to alternative methods (Table 2). In addition, a visual comparison makes it clear that, compared to FE meta-analysis, the MAMBA method tends to produce *less* significant p-values for SNPs with low ranks in the replication dataset (which are more likely to be spurious associations), but similar results for the higher-ranking SNPs (which are more likely to have true non-zero effects) (Fig. 4a). This demonstrates improved power and robustness for MAMBA. In contrast, the RE method can be underpowered, as many SNPs which are ranked highly in the replication cohort do not have significant *p*-values in the discovery cohort, which makes Kendall’s tau correlation coefficient lower (Fig. 4b). On the other hand, BE and RE2 methods tend to produce *p*-values similar to FE regardless of the replication rank of the SNP (Fig. 4c, d), suggesting that they may be sensitive to outliers and detect spurious associations as significant. Compared to MAMBA, MAMBA-est had a slight decrease in the concordance, as more noise was introduced as numerous correlated SNPs were fitted (Table 2).

**Table 2:** Kendall’s tau correlation of *p*-values between discovery meta-analysis and replication *p*-value. The highest correlations were marked by an asterisk.

		Phenotype		
Method	CigDay	DrnkWk	SmkCes	SmkInit
MAMBA	0.28*	0.29*	0.13*	0.37*
MAMBA-est	0.27	0.28	0.13*	0.36
FE	0.26	0.26	0.12	0.20
RE	0.17	0.12	0.01	0.09
RE2	0.25	0.27	0.12	0.13
BE	0.24	0.27	0.10	0.09

**Fig. 5:**
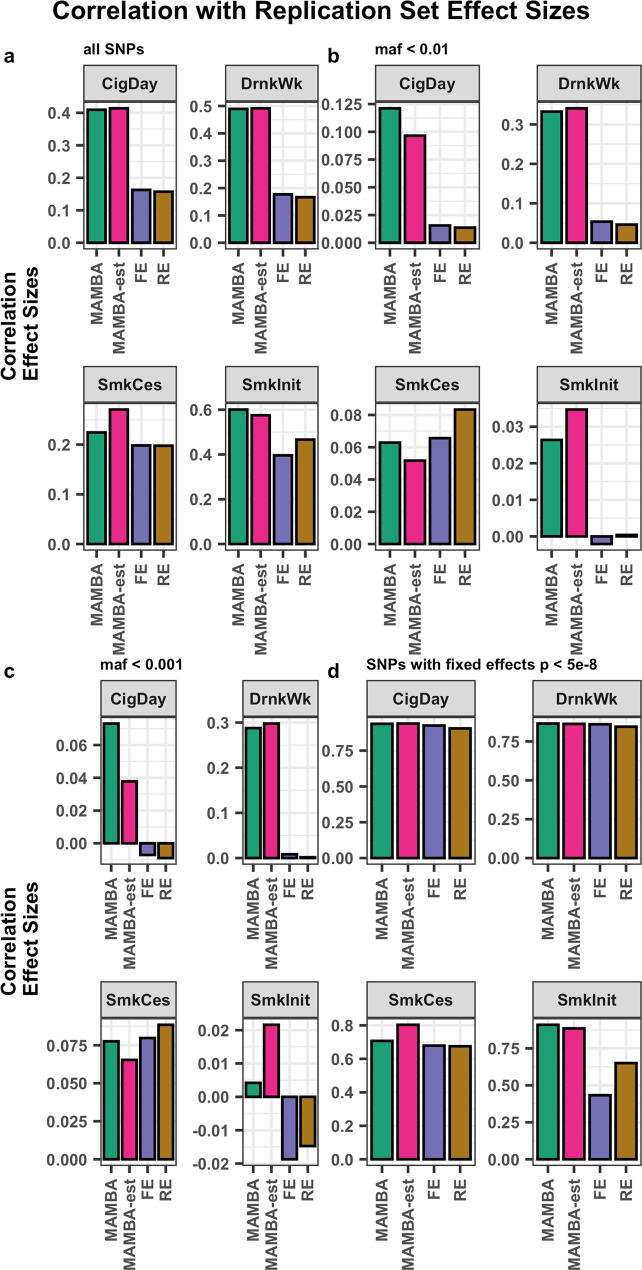
Pearson Correlation of meta-analysis estimated effect sizes with replication cohort effect sizes. We separately considered the correlation for (**a**) all variants (**b**) low-frequency variants (MAF < 0.01) (**c**) rare variants (MAF < 0.001) (**d**) genome-wide significant SNPs (two-sided unadjusted *p*-value < 5 × 10^−8^). Source data are provided as a Source Data file.

The MSE and Pearson correlation coefficient between discovery and replication cohort effect sizes were improved for all phenotypes and for practically every comparison considered, in particular for the genetic effect estimates for low and rare frequency variants with MAF < 1% (Supplementary Data 12). For example, low-frequency variant correlation (defined here as MAF < 1%) was improved from ~0.05 for FE and RE methods to 0.33 using the MAMBA method for the DrnkWk phenotype, along with a greater than 5-fold reduction in MSE. For the CigDay phenotype, correlation was improved from ~0.01 to 0.12 using the MAMBA method, along with a greater than 6-fold reduction in MSE (Fig. 5 and Supplementary Data 13). We plotted the estimated effect sizes from the FE and MAMBA method against the replication effect size estimates to demonstrate the improvement and shrinkage applied for each GSCAN phenotype (Supplementary Fig. 1). MAMBA-est had either nearly equal or slightly improved concordance and MSE with the replication dataset at the same pruned set of SNPs as the MAMBA method. This indicates that composite likelihood using information shared across SNPs in LD may in some cases benefit effect-size estimation compared to the LD pruned model. The agreement in the estimated outputs of the MAMBA and MAMBA-est models was high overall, with high correlations in both PPR (Pearson *ρ* = 0.85, Spearman *ρ* = 0.875), and estimated *P*-values (Pearson *ρ* = 0.92, Spearman *ρ* = 0.76) between MAMBA and MAMBA-est.

Evidence also suggests that SNPs identified by MAMBA have improved rate of replication in the 23andMe dataset, and this improvement is consistent at different replication significance thresholds (Supplementary Data 14).

#### MAMBA identifies outliers and non-replicable associations

Using MAMBA model outputs, we summarize the predicted number of outliers at each SNP and across GSCAN phenotype and MAF ranges (Table 3). We observed an increase in the predicted number of outliers for rare variants (MAF < 0.1%) compared to more common variants across phenotypes. For some traits, such as SmkInit, false positive associations may be pervasive prior to standard quality control procedures, and were detected even for common frequency variants (MAF > 1%). Among 2274 SNPs with suggestive evidence of association (i.e. *p* < 1 × 10^−5^), 87 SNPs had MAMBA PPR less than 0.1 (This includes 6, 7, and 74 loci from the CigDay, DrnkWk, and SmkInit phenotypes) (Supplementary Data 15). We made a Manhattan plot for detected SNPs with low PPR for the SmkInit phenotype and also highlighted SNPs within 1 MB of each detected non-replicable SNP (Supplementary Fig. 2). We see that in several cases, SNPs in LD with the detected outlier SNP are also significant, and form a misleading “peak” in the Manhattan plot typically indicative of a strong clear signal. Other outlier SNPs do not have significant SNPs in LD, thus may be challenging to judge for authenticity by visual inspection of the Manhattan plot. In addition, replicable rare-variant associations will inherently have fewer SNPs in LD, which would make visual judgement challenging. When examined in the replication data from the 23andMe cohort, only 4 of these 87 variants with low MAMBA PPR were replicated at a nominal significance threshold of *p* < 0.05, and 39 of these SNPs which were measured in the replication cohort have effect size estimates in the opposite direction of the discovery sample. Surprisingly, 25 of these SNPs for the SmkInit phenotype have reached genome-wide significance in the discovery cohort using a fixed effect meta-analysis. (See Supplementary Fig. 3 and Supplementary Data 15 for a description of detected non-replicable SNPs). On the other hand, among the 986 SNPs with estimated PPR >99%, 47% were nominally significant with *p* < 0.05 in the replication cohort 23andMe, and 79% with consistent direction of effects. Clearly, our comparison showed that MAMBA is very effective filtering out non-replicable signals, which we found to generally occur more frequently as MAF decreases. At the same time, it can recover many replicable low and rare frequency variant effects, which may be filtered out under more stringent quality control criteria (e.g. removing all variants with MAF<0.1% or with imputation *R*^2^<0.3). MAMBA thus can maximize the utility of the imputation-based GWAS, in particular for the discovery of associated lower frequency variants.

**Table 3:** Estimated Number of Outliers Statistics and Non-replicable SNPs in the GSCAN Dataset.

Phenotype	MAF range	Number of significant SNPs analyzed (*P* < 1 × 10^−5^)	Total expected # of outlier statistics^a^	Total expected # of Non-replicable SNPs^b^	Average of average # of outliers per study^c^	Maximum # outliers at each SNP	Median # of outliers at each SNP
SmkInit	0 < MAF < 0.001	306	62.83	64.85	0.026	2.151	0.007
SmkInit	0.001 < MAF < 0.01	87	22.9	22.73	0.027	1.027	0.015
SmkInit	0.01 < MAF < 0.5	301	6.09	4.63	0.027	2.173	0
SmkCes	0 < MAF < 0.001	424	26.09	22.62	0.046	1.349	0.043
SmkCes	0.001 < MAF < 0.01	100	5.01	3.93	0.043	0.276	0.04
SmkCes	0.01 < MAF < 0.5	201	7.09	4.81	0.045	0.252	0.025
DrnkWk	0 < MAF < 0.001	229	12.72	17.42	0.041	0.874	0.013
DrnkWk	0.001 < MAF < 0.01	74	0.81	0.71	0.042	0.051	0.01
DrnkWk	0.01 < MAF < 0.5	151	0.91	0.73	0.042	0.04	0.003
CigDay	0 < MAF < 0.001	203	16.2	19.86	0.042	0.817	0.025
CigDay	0.001 < MAF < 0.01	61	1.12	0.96	0.038	0.104	0.012
CigDay	0.01 < MAF < 0.5	137	1.27	0.99	0.04	0.1	0.002

Using estimated hyperparameters from MAMBA, we estimated the number of non-replicable SNPs and outlier statistics in each study as a way to quantify the extent of outlier statistics in real data.

^a^The total expected number of outlier statistics is calculated by $\mathop {\sum}\nolimits_j {[ {\widehat {\Pr }\left( {R_j = 0} \right)\mathop {\sum}\nolimits_k {\widehat {\Pr }\left( {O_{jk} = 1} \right)} } ]}$.

^b^The total expected number of non-replicable SNPs is calculated by $\mathop {\sum}\nolimits_j {\widehat {\Pr }\left( {R_j = 0} \right)}$.

^c^The average number of outliers per study is calculated by the total number of outliers divided by the number of studies that contributed to the meta-analysis.

#### Improved robust modeling of rare variants

The promising results from simulation and real data analysis encouraged us to reanalyze the GSCAN data using all available studies including 23andMe. We leveraged MAMBA to determine replicable and non-replicable signals without imposing any preset filtering criteria.

We first examined the replicability of the 556 reported hits (MAF > 0.1%) in the original GSCAN study, where we found 555 signals have PPR>99%. We identified rs79631993 to have low probability of replicability for the SmkInit phenotype (PPR = 0.08, MAMBA PVALUE=0.2). This SNP was highly significant as an outlier in one cohort, but became insignificant when meta-analyzed using the rest of the cohorts (Fixed Effects PVALUE=0.6).

Next, we explored if MAMBA can identify additional rare frequency (MAF < 0.1%) association signals which may be functionally important but were not identified in the original analyses. For GSCAN phenotypes, 4337 rare variants with MAF < 0.1% were analyzed, of which 2807 had PPR greater than 99%. We used the Ensembl Variant Effect Predictor^28^ to determine potential effects of these variants on genes and transcript sites, and found 262 SNPs which may function as either stop-gain or missense mutations, or are intronic mutations with genome-wide significant *p*-values (*P*_MAMBA_ < 5 × 10^−8^) (Supplementary Data 16).

We subsequently checked whether these associations were related to terms of “Alcoholism”, “Alcohol Drinking”, “Smoking”, “Tobacco Use Disorder”, and “Substance-Related Disorders” using PheGenI Phenotype-Genotype Integrator^29^. We found that 39 of the 262 SNPs corresponded to genes with previously cited associations for another smoking–drinking trait, with 5 being associated with both smoking and drinking phenotypes^30–36^ (*GRM5*, *PCDH9*, *CDH13*, *DPP6*, *ESR1*) (Supplementary Data 16). This highlighted the pervasive pleiotropy of rare variants for smoking and drinking addiction.

Among the 262 identified variants, a number of them are rare coding variants that point to genes with relevant mechanisms in addiction. The SNPs (rs121908486 and rs140272400) function as missense mutations, and reside in known lipids-associated genes (*SLC7A9* and *LIPC*). rs121908486 is a known pathogenic variant for the *SLC7A9* gene, and is identified as replicable for both DrnkWk (*P*_*MAMBA*_ < 7.6 × 10^−7^) and SmkCes (*P*_MAMBA_ < 3 × 10^−8^) phenotypes. *SLC7A9* is located within “amino acid transport across the plasma membrane” pathway which has also been associated with alcohol dependence^37^. A missense variant (rs28936679) in the *AANAT* gene is significantly associated with SmkCes (*P*_MAMBA_ < 3 × 10^−8^), and moderately associated with SmkInit (*P*_MAMBA _< 1.39 × 10^−7^). *AANAT* is involved in melatonin synthesis and controlling night/day rhythm in melatonin production. Mediation of circadian rhythm-driven mechanisms and synthesis of melatonin through *AANAT* expression has been proposed as an influential mechanism for cocaine and potentially other drug addictions^38^.

## Discussion

In this article, we presented a model-based method, MAMBA, for identifying non-zero replicable signals from a GWAMA and refining genetic effect estimates. We demonstrated using simulated and real datasets that MAMBA is capable of identifying non-replicable SNPs with high accuracy, and the refined effect size estimates from MAMBA have smaller MSE and are more concordant with estimates from independent datasets.

There are some existing methods for assessing the replicability of GWAS results^18,19^, which seek to identify the studies with non-zero genetic effects. However, because most of the genetic effects identified in GWAMA are small, statistical power to identify associations from each participating study is often limited, as evidenced by the low power of the SCREEN method. In contrast, our method focuses on quantifying whether the aggregated genetic effect in meta-analysis is non-zero, leveraging the strength and consistency of association signals between contributing studies and consequently leading to improved power and robustness.

Our approach implicitly assumes that the genetic effects for *genuine* association signals are relatively homogeneous. Though this assumption may be violated in practice, our simulations based upon the random effect model with considerable heterogeneity showed that the method still yields well-calibrated*p*-values, demonstrating the robustness of the method. For most identified genetic variants from GWAS, genetic heterogeneity for genuine association has typically been shown to be small^6,39^, particularly for studies that use only European samples. Currently, there is limited knowledge on the genetic heterogeneity in multi-ethnic studies that involve non-European samples, as a majority of existing large-scale genetic studies were based upon samples of European ancestry. In practice, the genetic effect heterogeneity may depend on the extent of gene by environment interaction, on whether the causal variant has different frequencies between populations, or on differences in linkage disequilibrium between ancestries. When multi-ethnic studies are considered, MAMBA can be applied to analyze each ancestry separately if there is strong evidence suggesting between-ancestry genetic effect heterogeneities.

In real data analysis of addiction phenotypes, we found MAMBA outperforms conventional heuristic quality control procedures that are being used in GWAS studies, such as examining if a GWAS “peak” has a strand of neighboring variants in LD which are also significantly associated. As we showed in the results, some spurious association signals also have supporting neighbors, which would likely be missed by visual inspection but were correctly pinpointed by MAMBA. We also found that our method can reliably identify replicable low-frequency SNPs and improve the coverage of imputation-based GWAMA to lower frequency variants. In practice, imputation-based GWAS meta-analyses often remove all low-frequency variants (i.e. MAF<0.1% or imputation quality R^2^ < 0.3) to protect against false positives. However, many of the low-frequency SNPs may still provide valuable association information. For future studies, we suggest using a more lenient filtering criteria in combination with PPR estimated by MAMBA to identify replicable associations, as current procedures for filtering variants may be overly conservative but can still fail to filter out spurious association signals.

The MAMBA model was developed to assess the replicability for the sentinel variants. When there are multiple independent signals in a locus, conditional analysis can be applied by first adjusting for the association signals from the top variant. The conditional p-values and effect sizes can be used as input for assessing the replicability for secondary signals. We also developed an extension to MAMBA called MAMBA-est, which extends MAMBA in a composite likelihood framework and can analyze correlated SNPs in each locus. A major application of MAMBA-est is to obtain more robust marginal effect size estimates for SNPs across the genome, which may be utilized in a variety of downstream analyses and in conjunction with other methods which take summary statistics as input, such as PrediXcan^40^ or LD Score regression^41^. When the interest is to assess the replicability of sentinel variants, MAMBA should be used instead of MAMBA-est, as it yields slightly more accurate estimates of the posterior probability of replicability.

Similar to the other meta-analysis methods we compared in this paper, MAMBA implicitly assumes that summary statistics from contributing studies are independent. General methodology has been proposed for decoupling the summary statistics from GWAS when there are overlapping subjects across studies^42^. These methods can be applied before assessing replicability with MAMBA. Extensions of MAMBA to overlapping subjects in meta-analysis is also a promising area of future research.

As the sequencing and genotyping cost continues to decrease, more genetic datasets will be generated and analyzed, and more studies will probe rare variants and variants with small genetic effects. Given the difficulty of finding a sufficiently sized replication cohort that is powerful enough to validate rare variant and small effects, model-based assessment of replicability in GWAMA should be seriously considered. We expect our method MAMBA will be a very useful tool for this purpose.

## Methods

### Model details

MAMBA is a hierarchical mixture model, which takes the SNP effects and their standard errors from participating studies as input. We define ${\boldsymbol{b}}_{\boldsymbol{j}} = ( {b_{j1}, \ldots ,b_{jK}} )^{\rm{T}}$ and ${\boldsymbol{s}}_{\boldsymbol{j}} = ( {s_{j1}, \ldots ,s_{jK}})^{\rm{T}}$, where *b*_*jk*_ and *s*_*jk*_ are the genetic effect estimate and standard error for SNP *j* in study *k*. We further use ${\boldsymbol{b}} = \left( {{\boldsymbol{b}}_1, \ldots ,{\boldsymbol{b}}_{\boldsymbol{M}}} \right)^T$ denote the effect size estimates for all *M* SNPs analyzed in the model.

In the MAMBA mixture model, we use the latent variable *R*_*j*_ to model whether SNP *j* has real non-zero effects, and the latent variable *O*_*jk*_ to denote whether a null SNP is a spuriously associated outlier in some studies. Replicable SNPs are assumed to have underlying marginal effect sizes *μ*, which follows a normal distribution with mean 0 and variance *τ*^2^. The proportion of replicable non-zero effect SNPs is denoted as *π*. The effect estimates for outlier SNPs is assumed to follow a normal distribution with inflated variance, and the proportion of outlier summary statistics for non-replicable zero-effect SNPs is denoted as *λ*.

Together, the distribution for the summary statistics *b*_*jk*_ follows

2 $$\begin{array}{*{20}{c}} {b_{jk}|R_j,O_{jk},\mu _j = \left\{ {\begin{array}{*{20}{c}} {\begin{array}{*{20}{l}} {N\big( {\mu _j,s_{jk}^2} \big),} \hfill & {R_j = 1} \hfill \\ {N\big( {0,\alpha s_{jk}^2} \big),} \hfill & {R_j = 0,O_{jk} = 1} \hfill \end{array}} \\ {N\big( {0,s_{jk}^2} \big),R_j = 0,O_{jk} = 0} \end{array}} \right.} \end{array},$$

where *μ*_*j*_ ~ *N*(0,*τ*^2^), *R*_*j*_ ~ Bernoulli (*π*), *O*_*jk*_ ~ Bernoulli (*λ*)

The hyperparameters of the model are denoted by *θ* = (*τ*^2^, α, *π*, *λ*), among which α is used to model the inflated effect sizes for “outlier” summary statistics.

Here, we assume that the contributed studies in a meta-analysis are non-overlapping and independent of each other, so the probability density function for a SNP *j* is

3 $$	p( {{\boldsymbol{b}}_{\boldsymbol{j}}}) = \pi \bigg[\int_{-\infty}^{\infty} {p\big(\mu_j|R_j=1\big)} \mathop { \prod }\nolimits_{k = 1}^K {p\big( {b_{jk}{\mathrm{|}}\mu_j,R_j = 1 } \big)d\mu_j\bigg] + \left( {1 - \pi } \right)} \\ 	\mathop {\prod}\nolimits_{k = 1}^K {\left[ {\lambda p\big( {b_{jk}{\mathrm{|}}R_j = 0,O_{jk} = 1} \big) + \left( {1 - \lambda } \right)p\big( {b_{jk}{\mathrm{|}}R_j = 0,O_{jk} = 0} \big)} \right]}$$

As pruned SNPs are independent of each other, the joint likelihood satisfies:

4 $$P\left( {\boldsymbol{b}} \right) = \mathop {\prod }\limits_{j = 1}^M p( {{\boldsymbol{b}}_{\boldsymbol{j}}} )$$

In fact, the likelihood in (4) can also be viewed as a composite likelihood when used to analyze genome-wide correlated SNPs and improve accuracy of genetic effect estimates (i.e. MAMBA-est).

We fit the joint model in (2) using an empirical Bayes approach, and estimate the hyperparameters *θ* = (*τ*^2^, α, *π*, *λ*) with an Expectation and Maximization (EM) algorithm (See Supplementary Note for details). The resulting estimated parameters are denoted as $\hat \pi ,\hat \tau ^2,\hat \lambda$, and $\hat \alpha$. While the likelihood and EM algorithm used to estimate both MAMBA and MAMBA-est models are the same, the hyperparameter estimates may not be comparable between the two models. This is because different sets of input summary statistics are provided for MAMBA and MAMBA-est. Given that our primary interest is to assess replicability and improve genetic effect estimates, the hyperparameters may be considered as nuisance parameters.

The posterior probability of a SNP having replicable effect (PPR) is estimated by

5 $$	\hat P(R_j =\, 1|{\boldsymbol{b}}_{\boldsymbol{j}}) = \frac{{\hat P({\boldsymbol{b}}_{\boldsymbol{j}}|R_j = 1)\hat P(R_j = 1)}}{{\hat P({\boldsymbol{b}}_{\boldsymbol{j}}|R_j = 1)P(R_j = 1) + \hat P({\boldsymbol{b}}_{\boldsymbol{j}}|R_j = 0)\hat P(R_j = 0)}}\\ 	 = \frac{{\hat \pi \left[ {{\int}_{ - \infty }^\infty P \big( {{\boldsymbol{b}}_{\boldsymbol{j}}{\mathrm{|}}R_j = 1,\mu _j,\hat \tau ^2} \big)P\big( {\mu _j{\mathrm{|}}\hat \tau ^2, R_j=1} \big)d\mu _j} \right]}}{{\hat \pi \left[ {{\int}_{ - \infty }^\infty P \big( {{\boldsymbol{b}}_{\boldsymbol{j}}|R_j = 1,\mu _j,\hat \tau ^2} \big)P\big( {\mu _j|\hat \tau ^2, R_j=1} \big)d\mu _j} \right] + \left( {1 - \hat \pi } \right)\mathop {\prod }\nolimits_{k = 1}^K \left[ {\widehat \lambda N\big(b_{jk},0,\hat \alpha s_{jk}^2\big) + (1 - \hat \lambda )N\big(b_{jk};0,s_{jk}^2\big)} \right]}}$$

and the posterior mean effect size for SNP *j* can be derived as

6 $$\begin{array}{l}\hat \mu _j = \hat P(R_j = 1|{\boldsymbol{b}}_{\boldsymbol{j}})E(\mu _j|R_j = 1,{\boldsymbol{b}}_{\boldsymbol{j}})\\ \,\,\,\,\,\,\, = \hat P( {R_j = 1|{\boldsymbol{b}}_{\boldsymbol{j}}} )\frac{{\mathop {\sum }\nolimits_{k = 1}^{\mathrm{K}} b_{jk}/s_{jk}^2}}{{1/\hat \tau ^2 + \mathop {\sum }\nolimits_{k = 1}^{\mathrm{K}} 1/s_{jk}^2}}\end{array}$$

(See Supplementary Note for a detailed derivation.)

In practice, the contributed summary association statistics often contain missing data, and the level of missingness is often higher for lower frequency variants^43^. This can be due to the low imputation quality for some variants, or because different studies use slightly different reference panel for imputation and hence harbor slightly different variant sites. When a genetic variant *j* is missing from cohort *k*, we exclude the missing summary statistics from the likelihood. The resulting analysis will still be valid, as the missingness occurs independently of the phenotype.

### Connections to fixed effect meta-analysis and weighted least square meta-analysis

MAMBA has a few interesting connections with existing methods. First, when there are no outliers, conditional on the mean parameter *μ*_*j*_, the model is reduced to fixed effect inverse variance weighted meta-analysis method. In this case, the likelihood for the summary statistics becomes

$$p(b_{jk}|\mu _j)\sim N\big( {\mu _j,s_{jk}^2} \big)$$

Yet, unlike fixed effect meta-analysis, our method includes a prior on the parameter *μ*_*j*_, which allows us to borrow strength from different variant sites.

Secondly, when the summary statistic for a non-replicable SNP is an outlier, its effect size is assumed to follow a normal distribution with variance inflated by a factor of *a*, i.e.

$$p\big(b_{jk}|R_j = 0,O_{jk} = 1 \big) \sim N\big(0,\alpha s_{jk}^2\big)$$

This “inflated variance” model is similar to the assumption made by a weighted least square meta-analysis. Previous studies have shown that a weighted least square meta-analysis with “inflated variance” assumption works equally well as a random effect model when there is heterogeneity in the effect sizes^44^. It also performs better than fixed effects methods when the variance of the estimator may not be accurately estimated^44^. In our model, this modeling strategy also helps MAMBA produce robust meta-analysis results in the presence of outlier effect size estimates.

### Calculation of *P*-values based upon bootstraps

To facilitate the comparison of MAMBA and other frequentist meta-analysis methods, we also developed a parametric bootstrap method to empirically generate the null distribution for the PPR computed from MAMBA. We then calculate p-values by comparing the sample-based posterior probability with the simulated empirical distribution. Specifically, the procedure includes three steps as follows:

1. We first estimate model parameters from the data and obtain the PPR for each SNP. We denote the estimated hyperparameters by $\hat \theta = (\hat \pi,\hat \alpha ,\hat \tau ^2,\hat \lambda )$
2. Next, generate simulated datasets based upon the estimated hyperparameters $\hat \theta$ from the model in (1), and estimate the PPR for all SNPs in the simulated datasets. Specifically, for the *l*^*th*^ bootstrap dataset, we generate the SNP effects based upon the following hierarchical model:
  $$	b_{mk}^l|\mu _m^l,s_{mk},R_m^l,O_{mk}^l\sim I\left( {R_m = 1} \right)N\left( {\mu _m^l,s_{mk}^2} \right)\\ 	+ I\left( {R_m = 0,O_{mk} = 0} \right)N\left( {0,s_{mk}^2} \right) + I\left( {R_m = 0,O_{mk} = 1} \right)N\left( {0,\hat \alpha s_{mk}^2} \right),$$
  where
  $$\mu _m^l\sim N\left( {0,\hat \tau ^2} \right),\,m = 1, \ldots ,M$$
  $$R_m^l\sim {\mathrm{Bernoulli}}\left( {\hat \pi } \right),\,m = 1, \ldots ,M$$
  $$O_{mk}^l\sim {\mathrm{Bernoulli}}\,({\hat \lambda } ),\,m = 1, \ldots ,M,k = 1, \ldots ,K$$
  A total of *L* bootstrap datasets will be generated, and *M* denotes the number of SNPs used in the original model. The standard errors $s_m^l$ for a simulated SNP *m* in dataset *l* are generated by bootstrap sampling from the rows of $S_{M \times k}$, where each row of $S_{M \times k}$ is a vector of standard errors for a SNP from the original dataset.
3. The posterior probabilities of the simulated non-associated SNPs (*R*_*j*_ = 0) from all *L* bootstrap datasets form an empirical distribution under the null hypothesis of no association. Let $| {R_{H_0}^l} |$ denote the number of simulated non-associated SNPs in bootstrap dataset *l*. We can calculate the p-value for SNP *j* in the original dataset by $p_j = \frac{1}{L}\sum_{l = 1}^L \frac{1}{{| {R_{H_0}^l} |}}\sum_{R_m^l = 0} I( p( {R_j = 1{\mathrm{|}}b_j} ) \le p( {R_m^l = 1{\mathrm{|}}b_m^l}) )$, where *p*(*R*_*j*_ = 1|*b*_*j*_) is the PPR in the original dataset for SNP *j*, and $p( {R_m^l = 1|b_m^l} )$ is the estimated PPR from the null SNP *m* in the *l*^*th*^ simulated dataset.

### GSCAN datasets

We evaluated the proposed methods using the meta-analysis dataset from the GSCAN consortium^17^. Four smoking and drinking phenotypes were used, including

I. Smoking Initiation (SmkInit) is a binary trait that contrasts ever and never smokers. Ever smokers were defined as individuals who have smoked >99 cigarettes in their lifetime, which is consistent with the definition by the Centre for Disease Control^45^;
II. Cigarettes per day (CigDay) is a quantitative trait that measures the average number of cigarettes smoked per day by ever smokers;
III. Smoking cessation (SmkCes) is a binary trait that contrasts former vs current smokers.
IV. Drinks Per Week (DrnkWk) is a quantitative trait that measures the average number of drinks per day by regular drinkers.

Age of Initiation (AgeInit) was the only GSCAN consortium phenotype excluded from our analysis, as there were too few SNPs which surpassed genome-wide significance using fixed effects meta-analysis.

### Preprocessing Workflow for Analyzing GSCAN Dataset with MAMBA and MAMBA-est

Using MAMBA, we assess the replicability of a pruned set of sentinel variants. In addition to the significant sentinel variants, we include randomly pruned markers from a reference panel to ensure that both non-replicable and replicable associated SNPs are represented in the dataset and the model may be reliably estimated. We follow the steps below to prune the GSCAN summary statistics and prepare the data to fit the MAMBA model.

- Step 0: We first perform fixed-effect GWAS meta-analysis to identify loci of interest with suggestive evidence of association (p-value < 1 × 10^−5^).
- Step 1a: Prune variants with suggestive evidence of association using the “clumping” procedure implemented in Plink v1.9^25^. These are the SNPs of interest for which we seek to assess the presence of a replicable non-zero effect. plink –bfile refpanel –clump fixed_effects_meta_sumstats –clump-p1 1e-5 –clump-kb 500 –clump-r2 0.1
- Step 1b: Given that the significant SNPs from Step 1a all initially appear to have non-zero effect from a fixed effects meta-analysis, we incorporate summary statistics from an independent set of variants randomly pruned based upon a reference panel. These SNPs allow the non-replicable, zero-mean component of the MAMBA mixture model to be reliably estimated, plink –bfile refpanel –indep-pairwise 500 kb 1 0.1 –maf 0.01
- Step 2: Create the dataset used to fit the MAMBA model by combining randomly pruned variants with clumped variants with suggestive evidence of association. We removed any randomly pruned markers within 500 kb of a clumped variant to ensure that the set of SNPs used to fit the model are in linkage equilibrium.

When using MAMBA-est to refine estimates of genetic effects, no pruning steps are needed and correlated SNPs can be analyzed directly.

### Additional software

Many analyses were conducted using R with packages including Matrix^46^, data.table version 1.12.2^47^, gridExtra version 2.3^48^, cowplot version 0.9.4^49^, metafor version 2.0.0^50^, xtable version 1.8.2^51^, and ggplot2 version 3.0.0^52^. For analysis using RE2 and BE (binary effect) models, METASOFT software v2.0.1 was used^14,53^.

### Reporting summary

Further information on research design is available in the Nature Research Reporting Summary linked to this article.

## Supplementary information

Supplementary Information

Reporting Summary

Descriptions of Additional Supplementary Files

Supplementary Data 1-16

## Data Availability

The aggregated GSCAN summary association statistics can be found at https://genome.psych.umn.edu/index.php/GSCAN^55^ Source data are provided with this paper.

## Source data

Source Data

## Code availability

An R package implementing the proposed methods can be found at https://github.com/dan11mcguire/mamba^54^.

## References

[1] Khera AV, Kathiresan S (2017). Genetics of coronary artery disease: discovery, biology and clinical translation. Nat. Rev. Genet..

[2] Teslovich TM (2010). Biological, clinical and population relevance of 95 loci for blood lipids. Nature.

[3] Fuchsberger, C. et al. The genetic architecture of type 2 diabetes. *Nature* **536**, 41–47 (2016). 10.1038/nature18642 PMC5034897 27398621

[4] Schumacher FR (2018). Association analyses of more than 140,000 men identify 63 new prostate cancer susceptibility loci. Nat. Genet..

[5] Huyghe JR (2019). Discovery of common and rare genetic risk variants for colorectal cancer. Nat. Genet..

[6] Liu, D. J. et al. Exome-wide association study of plasma lipids in >300,000 individuals. *Nat Genet* **49**, 1758–1766 (2017). 10.1038/ng.3977 PMC5709146 29083408

[7] Cohen JC, Boerwinkle E, Mosley TH, Hobbs HH (2006). Sequence variations in PCSK9, low LDL, and protection against coronary heart disease. N. Engl. J. Med..

[8] Tg (2014). Loss-of-function mutations in APOC3, triglycerides, and coronary disease. N. Engl. J. Med..

[9] Huffman JE (2018). Examining the current standards for genetic discovery and replication in the era of mega-biobanks. Nat. Commun..

[10] Visscher PM (2017). 10 years of GWAS discovery: biology, function, and translation. Am. J. Hum. Genet..

[11] Willer CJ, Li Y, Abecasis GR (2010). METAL: fast and efficient meta-analysis of genomewide association scans. Bioinformatics.

[12] Higgins JP, Thompson SG (2002). Quantifying heterogeneity in a meta-analysis. Stat .Med..

[13] Han B, Eskin E (2011). Random-effects model aimed at discovering associations in meta-analysis of genome-wide association studies. Am. J. Hum. Genet..

[14] Han B, Eskin E (2012). Interpreting meta-analyses of genome-wide association studies. PLoS Genet..

[15] Zeng P (2015). Statistical analysis for genome-wide association study. J. Biomed. Res..

[16] Locke AE (2015). Genetic studies of body mass index yield new insights for obesity biology. Nature.

[17] Liu M (2019). Association studies of up to 1.2 million individuals yield new insights into the genetic etiology of tobacco and alcohol use. Nat. Genet..

[18] Heller R, Yaacoby S, Yekutieli D (2014). repfdr: a tool for replicability analysis for genome-wide association studies. Bioinformatics.

[19] Amar D, Shamir R, Yekutieli D (2017). Extracting replicable associations across multiple studies: Empirical Bayes algorithms for controlling the false discovery rate. PLoS Comput. Biol..

[20] Li Q, Brown JB, Huang H, Bickel PJ (2011). Measuring reproducibility of high-throughput experiments. Ann. Appl. Stat..

[21] Lee CH, Cook S, Lee JS, Han B (2016). Comparison of two meta-analysis methods: inverse-variance-weighted average and weighted sum of *z*-scores. Genomics Inform.

[22] von Hippel PT (2015). The heterogeneity statistic I(2) can be biased in small meta-analyses. BMC Med. Res. Methodol..

[23] IntHout J, Ioannidis JP, Borm GF, Goeman JJ (2015). Small studies are more heterogeneous than large ones: a meta-meta-analysis. J. Clin. Epidemiol..

[24] Guolo A, Varin C (2017). Random-effects meta-analysis: the number of studies matters. Stat. Methods Med. Res..

[25] Purcell S (2007). PLINK: a tool set for whole-genome association and population-based linkage analyses. Am. J. Hum. Genet..

[26] McCarthy S (2016). A reference panel of 64,976 haplotypes for genotype imputation. Nat. Genet..

[27] Kendall MGA (1938). New measure of rank correlation. Biometrika.

[28] McLaren W (2016). The ensembl variant effect predictor. Genome Biol..

[29] Ramos EM (2014). Phenotype-Genotype Integrator (PheGenI): synthesizing genome-wide association study (GWAS) data with existing genomic resources. Eur J Hum Genet.

[30] Argos M (2014). Genome-wide association study of smoking behaviours among Bangladeshi adults. J. Med. Genet..

[31] Olfson E, Bierut LJ (2012). Convergence of genome-wide association and candidate gene studies for alcoholism. Alcohol. Clin. Exp. Res..

[32] McGue M (2013). A genome-wide association study of behavioral disinhibition. Behav. Genet..

[33] Park SL (2015). Mercapturic acids derived from the toxicants acrolein and crotonaldehyde in the urine of cigarette smokers from five ethnic groups with differing risks for lung cancer. PLoS ONE.

[34] Schumann G (2016). KLB is associated with alcohol drinking, and its gene product beta-Klotho is necessary for FGF21 regulation of alcohol preference. Proc. Natl Acad. Sci. USA.

[35] Treutlein J (2009). Genome-wide association study of alcohol dependence. Arch. Gen. Psychiatry.

[36] Zanetti KA (2016). Genome-wide association study confirms lung cancer susceptibility loci on chromosomes 5p15 and 15q25 in an African–American population. Lung Cancer.

[37] Zuo L (2015). Gene-based and pathway-based genome-wide association study of alcohol dependence. Shanghai Arch Psychiatry.

[38] Uz T, Javaid JI, Manev H (2002). Circadian differences in behavioral sensitization to cocaine: putative role of arylalkylamine N-acetyltransferase. Life Sci..

[39] Wen, X. & Stephens, M. Bayesian methods for genetic association analysis with heterogeneous subgroups: from meta-analyses to gene-environment interactions. *Ann. Appl. Stat.* **8**, 176–203 (2014). 10.1214/13-AOAS695 PMC4583155 26413181

[40] Gamazon ER (2015). A gene-based association method for mapping traits using reference transcriptome data. Nat. Genet..

[41] Bulik-Sullivan BK (2015). LD Score regression distinguishes confounding from polygenicity in genome-wide association studies. Nat. Genet..

[42] Han B. (2016). A general framework for meta-analyzing dependent studies with overlapping subjects in association mapping. Hum. Mol. Genet.

[43] Jiang Y (2018). Proper conditional analysis in the presence of missing data: Application to large scale meta-analysis of tobacco use phenotypes. PLoS Genet..

[44] Stanley TD, Doucouliagos H (2017). Neither fixed nor random: weighted least squares meta-regression. Res. Synthesis Methods.

[45] Centers for Disease Control and Prevention (CDC (2008). Cigarette smoking among adults—United States, 2007. MMWR Morb. Mortal Wkly. Rep..

[46] Bates, D. & Maechler, M. Matrix: sparse and dense matrix classes and methods. R package version 0.999375-43. http://cran.r-project.org/package=Matrix (2010).

[47] Dowle, M., Srinivasan, A., Short, T. & Lianoglou, S. data. table: Extension of data. frame. R package version 1 (2017).

[48] Auguie, B., Antonov, A. & Auguie, M. B. Package ‘gridExtra’. Miscellaneous Functions for “Grid” Graphics (2017).

[49] Wilke CO (2016). cowplot: streamlined plot theme and plot annotations for ‘ggplot2’. CRAN Repos..

[50] Viechtbauer W (2010). Conducting meta-analyses in R with the metafor package. J. Satistical Softw..

[51] Dahl, D. B., Scott, D., Roosen, C., Magnusson, A. & Swinton, J. xtable: Export tables to LaTeX or HTML. R package version, 1–5 (2009).

[52] Wickham, H. *ggplot2: Elegant Graphics for Data Analysis* (springer, 2016).

[53] Han B, Eskin E (2011). Random-effects model aimed at discovering associations in meta-analysis of genome-wide association studies. Am. J. Hum. Genet..

[54] McGuire, D. https://github.com/dan11mcguire/mamba (2020).

[55] Liu, M. et al. Data Related to Association studies of up to 1.2 million individuals yield new insights into the genetic etiology of tobacco and alcohol use. *Nat. Genet.* **51**, 237–244 (2019). 10.1038/s41588-018-0307-5 PMC6358542 30643251

