# Supplementary material for: Model-based assessment of replicability for genome-wide association meta-analysis
Source: Nat Commun. 2021 Mar 30;12:1964. doi: 10.1038/s41467-021-21226-z (PMC8009871; doi:10.1038/s41467-021-21226-z)
Supplement: Supplementary file 1 — Supplementary Information [file 41467_2021_21226_MOESM1_ESM.pdf]

Supplementary Information

Supplementary Figures:

Supplementary Figure 1: Comparison in effect size estimates between MAMBA, FE, and replication dataset. We plotted the model estimated effect sizes from Fixed Effects and MAMBA models against the observed replication effect sizes for each GSCAN phenotype. The solid black line has an intercept of zero and slope equal to one. Source data are provided as a Source Data file.

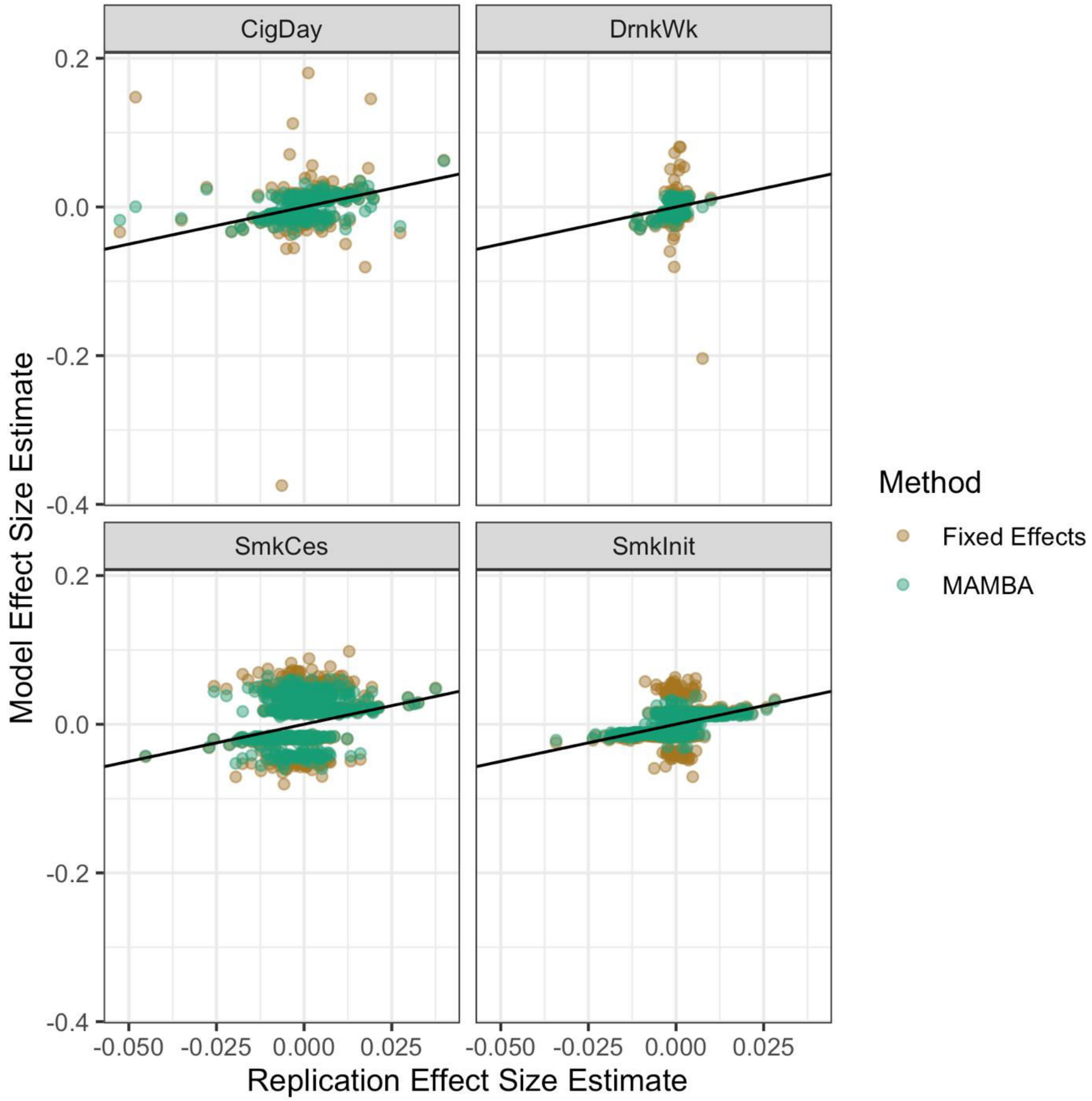

Supplementary Figure 2: **Detected Low PPR SNPs for SmkInit Phenotype**. Fixed effect p-values were plotted against the genomic position. Green cross indicates SNPs with posterior probability of replicability < 0.1, orange triangle indicates SNPs within +/- 1MB of an outlier SNP. For SNPs in chr14 and 15, MAMBA assigned low posterior probability of replicability. They were mostly driven by one study, and did not replicate in the 23andMe data. Yet, neighboring SNPs of these outliers have significant p-values. Ad-hoc procedures of GWAS quality control using "LD buddies" may not pick up this signal as outlier. The fixed-effects P-values displayed are two-sided and not adjusted for multiple comparisons.

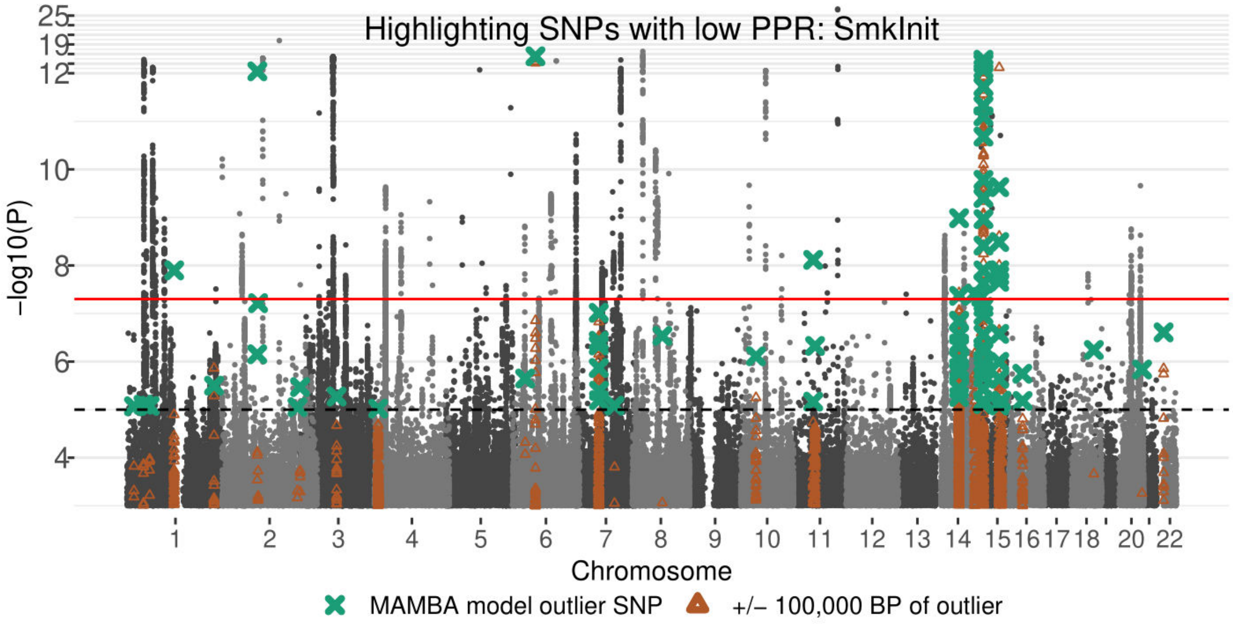

Supplementary Figure 3: **Three exemplary instances of non-replicable common variants from the discovery cohort (MAF>0.1%).** Cross mark indicates the inverse-variance weighted meta-analysis z-score from a two-sided hypothesis test, without any adjustment for multiple comparisons. Sizes of the dots are proportional to the sample size of the cohort. Orange color indicates the estimated posterior probability of a particular z-score to be an "outlier" given the association is non-replicable. Source data are provided as a Source Data file.

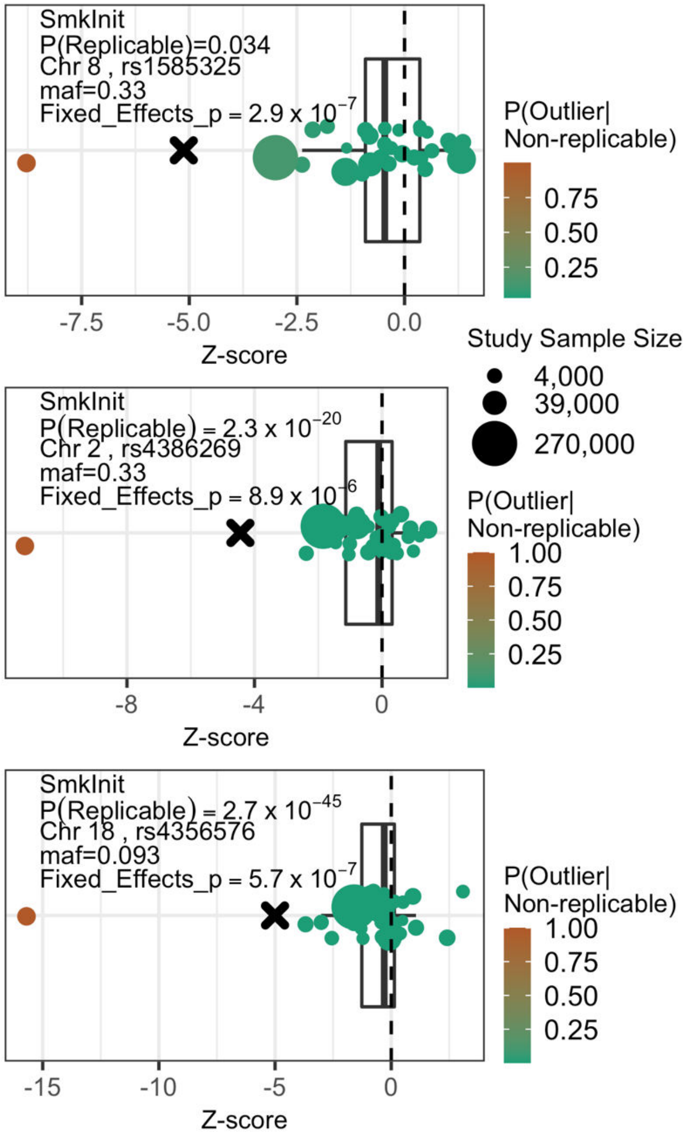

Supplementary Note
Methods Descriptions and Simulation Procedure for Summary Statistics

We simulate datasets assuming a particular data-generating process (DGP) for the summary statistics. Datasets of 50,000 markers are generated in two steps:

1. Simulate the true effect sizes from a spike-and-slab distribution, where the probability of a real-associated SNP is 0.01 and the variance of real-associated SNPs is $\tau^2 = 2.5 \times 10^{-4}$

$$\mu_j | R_j = \begin{cases} N(0, \tau^2), & R_j = 1 \\ 0 & R_j = 0 \end{cases}$$

2. Simulate the effect size estimates based upon the MAMBA, FE, RE, RE2, or BE DGP, conditional on the simulated true effect μ_j

The effect size estimate variances s_{jk}^2 are generated in our simulation by sampling with replacement from the variance of the observed genetic effects from GSCAN studies.

In each simulated dataset, the total sample size across cohorts is 500,000. We consider two combinations for the number of cohorts k being meta-analysed (5 or 10), and two combinations of sample size distribution (either equal or unequal sample sizes across different participating studies). We also vary the heterogeneity (I^2), effect size variance (τ^2), and variance inflation (α) depending on the DGP, as described below.

Description of Summary Statistic Simulation and Method

MAMBA Model

When summary statistics are generated according to the MAMBA model,

$$\beta_{jk} | R_j, O_{jk} \quad \sim \begin{cases} N(\mu_j, s_{jk}^2) & R_j = 1 \\ N(0, \alpha s_{jk}^2) & R_j = 0, O_{jk} = 1 \\ N(0, s_{jk}^2) & R_j = 0, O_{jk} = 0 \end{cases}$$

where $P(O_{jk} = 1) = 0.025$ indicating a small probability of an outlier. When a summary statistic is an outlier, the variance of the effect estimate is inflated by a factor of α. We set α to be either 2, 5, or 15 for each simulation dataset, representing increasing severity of the outliers.

Fixed Effect Meta-Analysis Method

Fixed effects meta-analysis assumes that the genetic effects are constant across different participating studies. The fixed effects meta-analysis statistic combines study level genetic effect estimates using weights that are inversely proportional to the variance of the genetic effect estimates, i.e.

$$Z_{IVW} = \frac{\sum_k b_k / s_k^2}{\sqrt{\sum_k 1 / s_k^2}}$$

Under the FE DGP, summary statistics are generated according to the fixed effects model,

$$b_{jk}|\mu_j \sim N(\mu_j, s_{jk}^2)$$

The underlying genetic effect sizes are simulated based upon $\mu_j \sim N(0, \tau^2)$. We set τ^2 to be either $5 \times 10^{-5}, 2.5 \times 10^{-4}, \text{or } 5 \times 10^{-4}$ for simulation datasets generated according to a fixed effects DGP.

Random Effects Meta-Analysis

Random effects methods assume that the underlying genetic effect varies across studies and follows a normal distribution. This is often written as a multi-level model, for example

$$b_{jk}|\eta_{jk} \sim N(\eta_{jk}, s_{jk}^2)$$

$$\eta_{jk}|\mu_j \sim N(\mu_j, \omega_j^2)$$

where η_{jk} is the study-specific genetic effect in cohort k, μ_j is the overall population mean genetic effect, and ω_j^2 is the variance of study-specific effects, characterizing heterogeneity across study cohorts. In addition to estimating the degree of heterogeneity through ω_j^2, random effects meta-analysis methods typically calculate a p-value testing the null hypothesis that the population mean effect is zero, i.e. $H_0: \mu_j = 0$. Under the multilevel random effect structure above, the meta-analysis test statistic can be calculated as

$$Z_j = \hat{\mu}_j \,/\, SE(\hat{\mu}_j)$$

$$\hat{\mu}_j = \frac{\sum_{j=1}^{k_j} \frac{b_{jk}}{s_{jk}^2 + \widehat{\omega}_j^2}}{\sum_{j=1}^{k_j} \frac{1}{s_{jk}^2 + \widehat{\omega}_j^2}}$$

$$SE(\hat{\mu}_j) = \sqrt{\sum_{j=1}^{k_j} \frac{1}{s_{jk}^2 + \widehat{\omega}_j^2}}$$

The DerSimonian and Laird method (1986) is one of the most popular random-effect methods, as it is easily calculated in closed form. The variance of study-specific effects is calculated as

$$\widehat{\omega}_j^2 = \max(0, \frac{Q_j - (k_j - 1)}{\sum_{j=1}^{k_j} 1/s_{jk}^2 - \frac{\sum_{j=1}^{k_j}(1/s_{jk}^2)^2}{\sum_{j=1}^{k_j} 1/s_{jk}^2}})$$

Where Q_j is Cochran's Q statistic[1]. We control the level of heterogeneity in simulation by setting the random effect variance ω_j^2 such that I^2 is either 0.05, 0.1, or 0.3 for all SNPs in a dataset, where $I^2 = 100\% \times \frac{(Q_j - k + 1)}{Q_j}$ and larger values of I^2 indicate higher levels of heterogeneity.

Han and Eskin's Random Effects Model (RE2)

Han and Eskin developed the $RE2$ test statistic as a likelihood ratio test[2], where effect heterogeneity only exists under the alternative hypothesis. In RE2, P-values are calculated from an empirical distribution of test-statistics obtained using simulations. Under this model, it's assumed that heterogeneity only exists under the alternative hypothesis, when $\mu_j \neq 0$.

$$\begin{aligned} \eta_{jk} | \mu_j, R_j \quad &\sim \begin{cases} N(\mu_j, \omega_j^2) & R_j = 1 \\ 0 & R_j = 0 \end{cases} \\ b_{jk} | \eta_{jk} \quad &\sim N(\eta_{jk}, s_{jk}^2) \end{aligned}$$

Similar to RE, we specify heterogeneity in our simulation through I^2. The random effect variance ω_j^2 is set such that I^2 is either 0.05, 0.1, or 0.3 for all SNPs in a dataset.

Han and Eskin's Binary Effects Model (BE)

Han and Eskin developed the Binary Effects (BE) model[3] as a meta-analysis model to interpret the study specific effect heterogeneity. In the BE model, for each SNP with real non-zero effects, we randomly sample a subset of the studies to have fixed effect size μ_j , while the rest of the studies have no effect. In simulations, both the RE2 and BE analyses were performed using METASOFT by the same authors with default parameters.

SCREEN Method:

SCREEN[4] is a method which was developed to identify the number of studies where a given SNP has replicable effects. SCREEN takes the p-values from each participating study as input. It calculates the posterior probability that a SNP is non-null in at least k studies, where k=$2,3,\ldots,\mathrm{K}$. In our simulation studies, we used the author's implementation of SCREEN available in the Supplement of their paper, and used the estimated FDR of $k = 2$ as the primary significance metric for a SNP. This calculates the FDR for "minimal replicability" in at least 2 studies, and thus maximizes the "power" for SCREEN in our comparisons.

EM Algorithm

Let $\Psi = (\pi, \lambda, \tau^2, \alpha)$, the parameters we need to estimate in our model. R_j and $(O_{j1}, \ldots, O_{jk_j})$ are the latent variables for SNP j, where $(R_j = 1)$ denotes at the SNP level the presence of a non-zero replicable effect. For non-replicable SNPs with zero-mean effect $(R_j = 0)$, we let O_{jk} denote at the cohort level whether a particular study's effect estimate b_{jk} is a misrepresentative outlier $(O_{jk} = 1)$, or a well-behaved estimate ($O_{jk} = 0$). The complete data log-likelihood is then given by

$$l^C(\Psi|(R, O, b, s^2)) =$$
$$\sum_{j=1}^{M}(R_j[\log(\pi) + \log p\big(b_j\big|R_j = 1)) +$$
$$\sum_{j=1}^{M}((1 - R_j)[\log (1 - \pi) +$$
$$\sum_{k=1}^{K} O_{jk} (\log (\lambda) + \log (p(b_{jk}|R_j = 0, O_{jk} = 1))) +$$
$$\sum_{k=1}^{K}(1 - O_{jk})(\log (1 - \lambda) + \log (p(b_{jk}|R_j = 0, O_{jk} = 0)))])$$

We fit the model by maximizing the expected complete data log likelihood at each iteration

E-step:

$$Q(\Psi, \Psi^{(t)}) \equiv E(l^C(\Psi)|b_j, s_j, \Psi^{(t)}) =$$
$$\sum_{j=1}^{M}(P(R_j = 1)[\log (\pi) + \log p\big(b_j\big|R_j = 1)) +$$
$$\sum_{j=1}^{M}(P(R_j = 0)[\log (1 - \pi)$$
$$+ \sum_{k=1}^{K} P\,(O_{jk} = 1|R_j = 0)(\log (\lambda) + \log (p(b_{jk}|R_j = 0, O_{jk} = 1)))$$
$$+ \sum_{k=1}^{K} P\,(O_{jk} = 0|R_j = 0)(\log (1 - \lambda) + \log (p(b_{jk}|R_j = 0, O_{jk} = 0)))])$$

Now we maximize $Q(\Psi, \Psi^{(t)})$. These solutions can be found by taking the derivative of $Q(\Psi, \Psi^{(t)})$ with respect to each parameter, and solving for the root.

$$\hat{R}_j^{(t)} \equiv$$
$$E(R_j | b_j, s_j, \Psi^{(t)})$$
$$= P(R_j = 1 | b_j, s_j, \Psi^{(t)})$$
$$= \frac{P(b_j | R_j = 1, \Psi^{(t)}) P(R_j = 1 | \Psi^{(t)})}{P(b_j | R_j = 1, \Psi^{(t)}) P(R_j = 1 | \Psi^{(t)}) + P(b_j, s_j | R_j = 0, \Psi^{(t)}) P(R_j = 0 | \Psi^{(t)})}$$
$$= \frac{\pi\, p^{(t)}(b_j | R_j = 1)}{\pi\, p^{(t)}(b_j | R_j = 1) + (1-\pi) \prod_{k=1}^{K} [\lambda^{(t)} p^{(t)}(b_{jk} | R_j = 0, O_{jk} = 1) + (1-\lambda^{(t)}) p^{(t)}(b_{jk} | R_j = 0, O_{jk} = 0)]}$$

$$\hat{O}_{jk}^{(t)} \equiv$$
$$E(O_{jk} | R_j = 0, b_j, s_j, \Psi^{(t)})$$
$$= P(O_{jk} = 1 | R_j = 0, b_j, s_j, \Psi^{(t)})$$
$$= \frac{\lambda^{(t)} p^{(t)}(b_{jk} | R_j = 0, O_{jk} = 1)}{\lambda^{(t)} p^{(t)}(b_{jk} | R_j = 0, O_{jk} = 1) + (1-\lambda^{(t)}) p^{(t)}(b_{jk} | R_j = 0, O_{jk} = 0)}$$

M-step:

$$\hat{\pi}^{(t+1)} = \frac{\sum_{j=1}^{M} \hat{R}_j^{(t)}}{M}$$

$$\hat{\lambda}^{(t+1)} = \frac{\sum_{j=1}^{M} (1 - \hat{R}_j^{(t)}) \sum_{k=1}^{K} \hat{O}_{jk}^{(t)}}{\sum_{j=1}^{M} (1 - \hat{R}_j^{(t)}) K}$$

$$\hat{\alpha}^{(t+1)} = \frac{\sum_{j=1}^{M} (1 - \hat{R}_j^{(t)}) \sum_{k=1}^{K} \hat{O}_{jk}^{(t)} b_{jk}^2 / s_{jk}^2}{\sum_{j=1}^{M} (1 - \hat{R}_j^{(t)}) \sum_{k=1}^{K} \hat{O}_{jk}^{(t)}}$$

$$\hat{\tau}^{2(t+1)} = argmax_{\tau^2} \sum_{j=1}^{M} \hat{R}_j^{(t)} [log(1/\tau^2) - log(\sum_{k=1}^{K} 1/s_{jk}^2 + 1/\tau^2) + \frac{\sum_{k=1}^{K} (\frac{b_{jk}^2}{s_{jk}^2})^2}{\sum_{k=1}^{K} 1/s_{jk}^2 + 1/\tau^2}]$$

The posterior mean effect size is given by

$$\mu_j^{(t+1)} = P(R_j = 1 | b_j, s_j, \Psi^{(t)}) E(\mu_j | R_j = 1, b_j, s_j)$$
$$= \hat{R}_j^{(t)} \frac{\sum_{k=1}^{K} b_{jk} / s_{jk}^2}{1/\hat{\tau}^{2(t)} + \sum_{k=1}^{K} 1/s_{jk}^2}$$

ADDITIONAL CONSORTIUM ACKNOWLEDGEMENTS

23andMe Research Team

Michelle Agee[1], Babak Alipanahi[1], Adam Auton[1], Robert K. Bell[1], Katarzyna Bryc[1], Sarah L. Elson[1], Pierre Fontanillas[1], Nicholas A. Furlotte[1], David A. Hinds[1], Bethann S. Hromatka[1], Karen E. Huber[1], Aaron Kleinman[1], Nadia K. Litterman[1], Matthew H. Mcintyre[1], Joanna L. Mountain[1], Carrie A. M. Northover[1], J. Fah Sathirapongsasuti[1], Olga V. Sazonova[1], Janie F. Shelton[1], Suyash Shringarpure[1], Chao Tian[1], Joyce Y. Tung[1], Vladimir Vacic[1], Catherine H. Wilson[1], Steven J. Pitts[1],

HUNT All-In Psychiatry

Amy Mitchell[3], Anne Heidi Skogholt[2], Bendik S. Winsvold[3,4], Børge Sivertsen[5,6,7], Eystein Stordal[6,8], Gunnar Morken[6,9], H˚avard Kallestad[6,9], Ingrid Heuch[7], John-Anker Zwart[3,4,10], Katrine Kveli Fjukstad[11,12], Linda M. Pedersen[3], Maiken Elvestad Gabrielsen[2], Marianne Bakke Johnsen[3,10], Marit Skrove[13], Marit Sæbø Indredavik[6,13], Ole Kristian Drange[6,9], Ottar Bjerkeset[6,14], Sigrid Børte[3,10], and Synne Øien Stensland[3,15]

[1]23andMe, Inc., Mountain View, California, USA
[2]K.G. Jebsen Center for Genetic Epidemiology, Department of Public Health and Nursing, Norwegian University of Science and Technology, Trondheim, Norway
[3]FORMI and Department of Neurology, Oslo University Hospital, Oslo, Norway
[4]Department of Neurology, Oslo University Hospital, Oslo, Norway.
[5]Department of Health Promotion, Norwegian Institute of Public Health, Bergen, Norway.
[6]Department of Mental Health, Faculty of Medicine and Health Sciences, Norwegian University of Science and Technology, Trondheim, Norway.
[7]Department of Research and Innovation, Helse-Fonna HF, Haugesund, Norway.
[8]Department of Psychiatry, Hospital Namsos, Nord-Trøndelag Health Trust, Namsos, Norway.
[9]Division of Mental Health Care, St. Olavs Hospital, Trondheim University Hospital, Trondheim, Norway.
[10]Institute of Clinical Medicine, University of Oslo, Oslo, Norway.
[11]Department of Psychiatry, Nord-Trøndelag Hospital Trust, Levanger Hospital, Levanger, Norway.
[12]Department of Laboratory Medicine, Children's and Women's Health, Norwegian University of Science and Technology, Trondheim, Norway.
[13]Regional Centre for Child and Youth Mental Health and Child Welfare, Department of Mental Health, Faculty of Medicine and Health Sciences, Norwegian University of Science and Technology, Trondheim, Norway.
[14]Faculty of Nursing and Health Sciences, Nord University, Levanger, Norway.
[15]Norwegian Centre for Violence and Traumatic Stress Studies, Oslo, Norway.

SUPPLEMENTARY REFERENCES

1. COCHRAN, W.G. THE COMPARISON OF PERCENTAGES IN MATCHED SAMPLES. *Biometrika* **37**, 256-266 (1950).
2. Han, B. & Eskin, E. Random-effects model aimed at discovering associations in meta-analysis of genome-wide association studies. *Am J Hum Genet* **88**, 586-98 (2011).
3. Han, B. & Eskin, E. Interpreting meta-analyses of genome-wide association studies. *PLoS Genet* **8**, e1002555 (2012).
4. Amar, D., Shamir, R. & Yekutieli, D. Extracting replicable associations across multiple studies: Empirical Bayes algorithms for controlling the false discovery rate. *PLoS Comput Biol* **13**, e1005700 (2017).
