# Supplementary material for: Model-based assessment of replicability for genome-wide association meta-analysis
Source: Nat Commun. 2021 Mar 30;12:1964. doi: 10.1038/s41467-021-21226-z (PMC8009871; doi:10.1038/s41467-021-21226-z)
Supplement: Supplementary file 2 — Reporting Summary [file 41467_2021_21226_MOESM2_ESM.pdf]

nature research

Corresponding author(s): Dajiang Liu, Qunhua Li, Bibo Jiang

Last updated by author(s): Nov 23, 2020

Reporting Summary

Nature Research wishes to improve the reproducibility of the work that we publish. This form provides structure for consistency and transparency in reporting. For further information on Nature Research policies, see our Editorial Policies and the Editorial Policy Checklist.

Statistics

For all statistical analyses, confirm that the following items are present in the figure legend, table legend, main text, or Methods section.

n/a	Confirmed	
☐	☒	The exact sample size (n) for each experimental group/condition, given as a discrete number and unit of measurement
☒	☐	A statement on whether measurements were taken from distinct samples or whether the same sample was measured repeatedly
☐	☒	The statistical test(s) used AND whether they are one- or two-sided<br>*Only common tests should be described solely by name; describe more complex techniques in the Methods section.*
☐	☒	A description of all covariates tested
☐	☒	A description of any assumptions or corrections, such as tests of normality and adjustment for multiple comparisons
☐	☒	A full description of the statistical parameters including central tendency (e.g. means) or other basic estimates (e.g. regression coefficient) AND variation (e.g. standard deviation) or associated estimates of uncertainty (e.g. confidence intervals)
☐	☒	For null hypothesis testing, the test statistic (e.g. F, t, r) with confidence intervals, effect sizes, degrees of freedom and P value noted<br>*Give P values as exact values whenever suitable.*
☐	☒	For Bayesian analysis, information on the choice of priors and Markov chain Monte Carlo settings
☒	☐	For hierarchical and complex designs, identification of the appropriate level for tests and full reporting of outcomes
☐	☒	Estimates of effect sizes (e.g. Cohen's d, Pearson's r), indicating how they were calculated

Our web collection on statistics for biologists contains articles on many of the points above.

Software and code

Policy information about availability of computer code

Data collection	Summary statistic files were generated using rvtests v20171009: https://github.com/zhanxw/rvtests.
Data analysis	An R package implementing the proposed methods can be found at https://github.com/dan11mcguire/mamba<br>Pruning and clumping using: PLINK v1.90b6.10 www.cog-genomics.org/plink/1.9/;<br>RE2 and BE methods applied using METASOFT v2.0.1;<br>MAMBA model fitting and simulation using : mamba R package https://github.com/dan11mcguire/mamba;<br>metafor R package version 2.0.0; Matrix R package version 1.2.15; ggplot2 R package version 3.0.0; xtable R package version 1.8.2;<br>RColorBrewer R package version 1.1.2; data.table R package version 1.12.2; gridExtra R package version 2.3; cowplot R package version 0.9.4

For manuscripts utilizing custom algorithms or software that are central to the research but not yet described in published literature, software must be made available to editors and reviewers. We strongly encourage code deposition in a community repository (e.g. GitHub). See the Nature Research guidelines for submitting code & software for further information.

Data

Policy information about availability of data

All manuscripts must include a data availability statement. This statement should provide the following information, where applicable:

- Accession codes, unique identifiers, or web links for publicly available datasets
- A list of figures that have associated raw data
- A description of any restrictions on data availability

The aggregated GSCAN summary association statistics can be found at https://genome.psych.umn.edu/index.php/GSCAN

Field-specific reporting

Please select the one below that is the best fit for your research. If you are not sure, read the appropriate sections before making your selection.

☒ Life sciences ☐ Behavioural & social sciences ☐ Ecological, evolutionary & environmental sciences

For a reference copy of the document with all sections, see nature.com/documents/nr-reporting-summary-flat.pdf

Life sciences study design

All studies must disclose on these points even when the disclosure is negative.

Sample size	All available cohorts from the initial GSCAN analysis were included, which maximized the potential sample size for this study. The study is one of the largest GWAS meta-analysis. At sample size N= l.2 million, we have 90% power for detecting associations of genetic variants with MAF=.25 and beta=0.01.
Data exclusions	We analyzed the published datasets of summary association statistics, and no contributed datasets were excluded.
Replication	We used one replicate for our replication. Specifically, we split our dataset into a discovery sample, and a replication sample. Discovery sample consists of all cohorts but 23andMe study, while the replication cohort consists of only the 23andMe study. Association signals identified in the discovery cohorts were replicated in the 23andMe study. In addition, our manuscript described a principled statistical approach to assess replicability in meta-analysis, which allows researchers to assess if a signal is genuine without a replication dataset.
Randomization	N/A. Our study only analyzed contributed summary association statistics, and no new data was collected.
Blinding	N/A. Our study only analyzed contributed summary association statistics, and no new data was collected.

Reporting for specific materials, systems and methods

We require information from authors about some types of materials, experimental systems and methods used in many studies. Here, indicate whether each material, system or method listed is relevant to your study. If you are not sure if a list item applies to your research, read the appropriate section before selecting a response.

Materials & experimental systems

n/a	Involved in the study
☒	☐ Antibodies
☒	☐ Eukaryotic cell lines
☒	☐ Palaeontology and archaeology
☒	☐ Animals and other organisms
☒	☐ Human research participants
☒	☐ Clinical data
☒	☐ Dual use research of concern

Methods

n/a	Involved in the study
☒	☐ ChIP-seq
☒	☐ Flow cytometry
☒	☐ MRI-based neuroimaging
