# Supplementary material for: Model-based assessment of replicability for genome-wide association meta-analysis
Source: Nat Commun. 2021 Mar 30;12:1964. doi: 10.1038/s41467-021-21226-z (PMC8009871; doi:10.1038/s41467-021-21226-z)
Supplement: Supplementary file 3 — Descriptions of Additional Supplementary Files [file 41467_2021_21226_MOESM3_ESM.pdf]

Descriptions of Additional Supplementary Files

Supplementary Data 1

Description: Simulation evaluation of type-I error.

Supplementary Data 2

Description: Model-specific cutoff levels used for calculating Power, where cutoffs correspond to empirical type-I error rate of 1×10^{-6} for each model under each DGP.

Supplementary Data 3

Description: Summary of MAMBA hyperparameter estimates under simulation.

Supplementary Data 4

Description: Additional Simulation Scenarios.

Supplementary Data 5

Description: Phenotypic Descriptive Statistics for Continuous Traits: Cigarettes per Day, Drinks per Week

Supplementary Data 6

Description: Phenotypic Descriptive Statistics for Binary Traits: Smoking Cessation, Smoking Initiation

Supplementary Data 7

Description: Summary of model computation time.

Supplementary Data 8

Description: Estimated Hyperparameters from MAMBA models for Cigarettes per Day phenotype.

Supplementary Data 9

Description: Estimated Hyperparameters from MAMBA models for Drinks per Week phenotype.

Supplementary Data 10

Description: Estimated Hyperparameters from MAMBA models for Smoking Initiation phenotype.

Supplementary Data 11

Description: Estimated Hyperparameters from MAMBA models for Smoking Cessation phenotype.

Supplementary Data 16

Description: Selected rare variants (MAF < 0.001) with PPR > 0.99.
